# A Rapid Adaptation Approach for Dynamic Air-Writing Recognition Using Wearable Wristbands with Self-Supervised Contrastive Learning

**DOI:** 10.1007/s40820-024-01545-8

**Published:** 2024-10-16

**Authors:** Yunjian Guo, Kunpeng Li, Wei Yue, Nam-Young Kim, Yang Li, Guozhen Shen, Jong-Chul Lee

**Affiliations:** 1 Department of Electronic Convergence Engineering, Kwangwoon University, Seoul, 01897 South Korea; 2 Radio Frequency Integrated Circuit (RFIC) Bio Centre, Kwangwoon University, Seoul, 01897 South Korea; 3 Department of Electronic Engineering, Kwangwoon University, Seoul, 01897 South Korea; 4 School of Microelectronics, Shandong University, Jinan, 250101 People’s Republic of China; 5 State Key Laboratory of Integrated Chips and Systems, Fudan University, Shanghai, 200433 People’s Republic of China; 6 School of Integrated Circuits and Electronics, Beijing Institute of Technology, Beijing, 100081 People’s Republic of China

**Keywords:** Wearable wristband, Self-supervised contrastive learning, Dynamic gesture, Air-writing, Human–machine interaction

## Abstract

Wearable wristband systems leverage deep learning to revolutionize hand gesture recognition in daily activities. Unlike existing approaches that often focus on static gestures and require extensive labeled data, the proposed wearable wristband with self-supervised contrastive learning excels at dynamic motion tracking and adapts rapidly across multiple scenarios. It features a four-channel sensing array composed of an ionic hydrogel with hierarchical microcone structures and ultrathin flexible electrodes, resulting in high-sensitivity capacitance output. Through wireless transmission from a Wi-Fi module, the proposed algorithm learns latent features from the unlabeled signals of random wrist movements. Remarkably, only few-shot labeled data are sufficient for fine-tuning the model, enabling rapid adaptation to various tasks. The system achieves a high accuracy of 94.9% in different scenarios, including the prediction of eight-direction commands, and air-writing of all numbers and letters. The proposed method facilitates smooth transitions between multiple tasks without the need for modifying the structure or undergoing extensive task-specific training. Its utility has been further extended to enhance human–machine interaction over digital platforms, such as game controls, calculators, and three-language login systems, offering users a natural and intuitive way of communication.

**Supplementary Information:**

The online version contains supplementary material available at 10.1007/s40820-024-01545-8.

## Highlights

- Utilizing self-supervised learning, the proposed wearable wristband with a four-channel sensing array and wireless transmission module is developed for tracking air-writing and dynamic gestures.
- The model can learn prior features from unlabeled signals of random wrist movements, significantly reducing the dependency on extensive labeled data for training.
- The wristband system rapidly adapts to multiple scenarios after fine-tuning using few-shot data, enhancing user interaction through natural and intuitive communication.

**Supplementary Information:**

The online version contains supplementary material available at 10.1007/s40820-024-01545-8.

## Graphical abstract

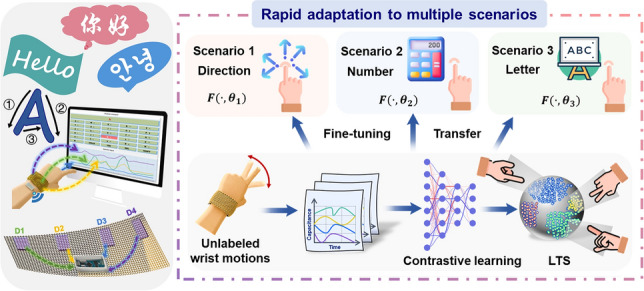

## Introduction

Gesture recognition, acknowledged as an intuitive and natural mode of communication, interprets intentional hand movements to convey significant information and has garnered substantial attention in the field of human–machine interaction [1–3]. Common techniques for capturing hand movements include image recognition, radar systems, and wearable technology [4–6]. Bulky devices such as high-resolution cameras, accelerometers, or radar systems are not suitable for daily wear [7–9]. In contrast, wearable devices offer an attractive alternative for monitoring hand movements and intentional gestures, as they can be seamlessly integrated into various accessories [10–12]. The choice of device placement significantly impacts both wearing comfort and the effectiveness of data acquisition [13, 14]. Given that most tendons and muscle groups responsible for hand movements are located beneath the wrist skin, wristbands offer an optimal placement option compared to positioning devices on the fingers or the back of the hand [15], providing high-sensitivity devices with the opportunity to precisely track subtle movements. The direct mapping of specific gestures has been widely recognized and developed. Wang et al. proposed a gesture recognition wristband by integrating a triboelectric nanogenerator and a piezoelectric nanogenerator, achieving a maximum accuracy of 92.6% in recognizing 26 letters [16]. Similarly, Wu et al. deployed seven triboelectric nanogenerator sensors into a smart wristband, successfully classifying 21 hand motions and enabling wireless control through air gestures [17]. These wristband-integrated systems enable static gesture recognition through specific finger gestures. However, the one-to-one mapping between specific gestures and information restricts the conveyable data, and excessive correspondence places a significant burden on the user. Therefore, there is an urgent need to develop intuitive mapping rules and recognition systems that align with user habits and cognitive processes.

Dynamic gestures based on air-writing utilize direct handwriting mapping rules for characters, which can optimize user experience and enhance information density [18–20]. Air-writing involves tracing letters or numbers by moving hands or fingers in free space to form a virtual text interface. It has proven valuable in scenarios where conventional writing methods are impractical, especially in translating sign language, improving experiences in augmented reality or virtual reality, and facilitating various gesture-driven interfaces [21–23]. For example, Liu et al. utilized a stretchable and conductive hydrogel strain sensor to recognize the entire dynamic process of air-writing 26 English letters [24]. This approach of recognizing multi-stroke characters significantly enhances the richness of dynamic information by tracking the spatial writing process while preserving the user’s natural habits [25].

However, the diversity in character forms and writing styles presents significant challenges for the recognition process. Recent advancements in deep neural networks for processing time-series or array data from wearable devices have opened up new possibilities for feature extraction and classification in complex tasks [26–28]. In combination with convolutional neural network algorithms, Li et al. designed a virtual text-entry interface using gloves integrated with unimodal sensors for finger air-writing applications involving letters and numbers [29]. Although these algorithms have the capability to analyze and learn gestures captured by wearable devices, they are constrained by conventional supervised learning methods, which heavily rely on extensive manually labeled data for single-task execution, making them both time-consuming and labor-intensive [30–33]. Adapting these systems to new users or incorporating new scenarios requires the collection of large amounts of new labeled data or modifications to the model architecture. This raises development and maintenance costs, and extends system deployment time, underscoring their limitations in generalizability and scalability. Consequently, improving data processing efficiency and optimizing learning and training procedures to enhance user experience in air-writing systems is a significant challenge.

**Fig. 1:**
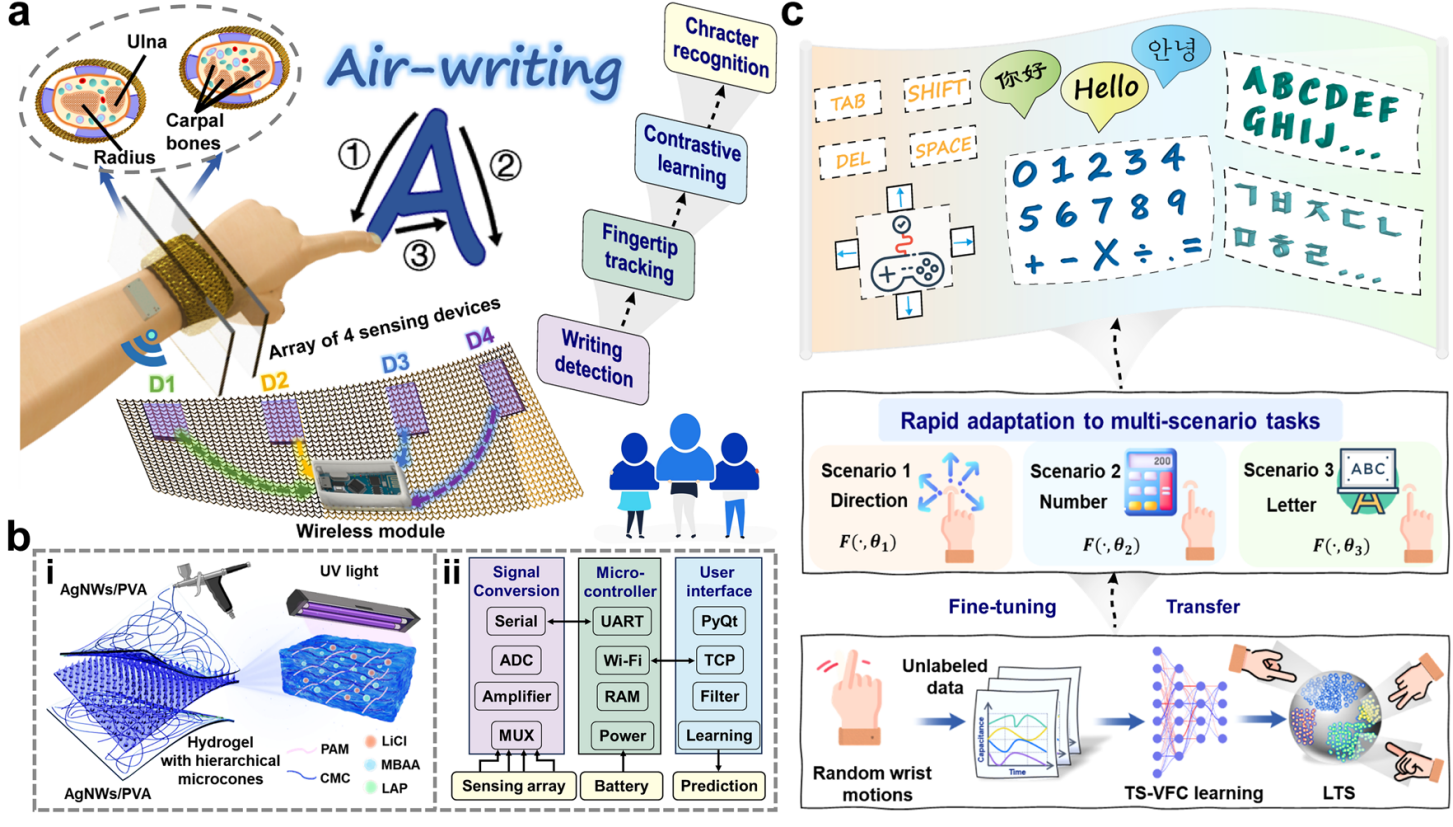
Illustration of the wearable wristband and air-writing prediction process. **a** Wristband containing four sensing devices (D1–D4) equipped with a wireless Wi-Fi module for air-writing recognition. **b** (i) Exploded view of the design layout of iontronic device, including AgNWs/PVA electrodes and photocurable ionic hydrogel with hierarchical microcones. (ii) Brief block diagram of the wristband system and the customized user interface. **c** Real-time prediction and display of air-writing through TS-VFC learning. Rapid adaptation process of directions, numbers, and letters: learning prior features from random wrist movements as LTS via TS-VFC learning, and fine-tuning with few-shot labeled data for rapid adaptation to diverse tasks

This study presents the development of a wearable fiber wristband designed for dynamic gesture recognition in human–computer interactions utilizing the time-series cross-view fusion contrastive (TS-VFC) learning algorithm, as depicted in Fig. 1. This general learning framework can rapidly adapt to multiple scenarios without requiring extensive labeled data collection. The wristband, meticulously designed to accommodate the anatomical and muscular dynamics of the wrist, features an array of four flexible iontronic devices, coupled with a Wi-Fi module for wireless communication, ensuring comfort during prolonged daily use. Each device consists of ultrathin electrodes (25 μm) made from silver nanowires (AgNWs)/improved polyvinyl alcohol (PVA) and a hydrogel dielectric layer with microcone structures (Fig. S1). Consequently, its output signal exhibits high sensitivity and precision in capturing subtle gestures. The TS-VFC learning method collects unlabeled random motion data from the wrist, creating a latent time space (LTS) that encapsulates prior features. It enables the model to be fine-tuned for new users or scenarios with few-shot labeled data, thus eliminating the need for extensive training or model redesign. Our experiments demonstrate that transfer learning, with just 5-shot labeled data, can achieve accuracy rates of up to 94.9%. This capability allows the pre-trained model to rapidly adapt to different gesture recognition tasks, including directional movements and air-writing of numbers and letters. Moreover, we showcased practical applications such as game control, calculator operation, and login systems, underscoring the practicality and potential for human–machine interaction. This wristband system effectively translates hand gestures into digital commands, making it a promising candidate to facilitate the interaction of diverse electronic interfaces.

## Experimental Section

### Materials

Acrylamide (AM, 99%), sodium carboxymethyl cellulose (CMC), N,N’-methylenebisacrylamide (MBAA, 99%), polyethylene glycol diacrylate (PEGDA), lithium chloride (LiCl, 99%), diphenyl(2,4,6-trimethylbenzoyl)phosphine oxide (TPO) and ethylene glycol (EG) were procured from Sigma-Aldrich. The photoinitiator used was lithium phenyl(2,4,6-trimethylbenzoyl) phosphinate (LAP) obtained from the Tokyo chemical industry, Tokyo, Japan. polydimethylsiloxane (PDMS) precursor and curing agent (Sylgard 184) were purchased from Dow Corning. AgNWs dispersions (DT-AGNW-N30-EOH) were supplied by Ditto Technology Co., Ltd. (South Korea). Improved PVA was purchased from Zhuzhou Shifeng New Blue Sky Materials Co., Ltd. (China). All chemical reagents were analytical grade and used without further purification.

### Preparation of Iontronic Device

The device was fabricated by following the procedure outlined in Fig. S1. Initially, a resin mold featuring hierarchical microcones was created using a Stereolithography 3D printer (Formlabs Form 3). Subsequently, the PDMS precursor and curing agent were coated onto the mold at a weight ratio of 10:1 and cured at 80 °C for 1 h. The resulting cured PDMS, with complementary patterns, was then peeled off and utilized as the second mold for the hydrogel. The hydrogel was prepared using AM as the monomer, CMC as the nanofiller, MBAA and PEGDA as crosslinkers, LiCl as conductive salts, and TPO as the photo-initiator. Specifically, the AM (16 wt%), CMC (2 wt%), MBAA (0.3 wt%), PEGDA (0.3 wt%), EG (5 wt%), and TPO (0.1 wt%) were dissolved in a 6 M LiCl aqueous solution at room temperature. Both AM and CMC were used for the hydrogel polymer networks, and the inclusion of EG was beneficial for enhancing the dehydration resistance. EG not only protects the polymer interactions by forming stable ion clusters with Li^+^, effectively reducing the hydration of the lithium salt, but also enhances the mechanical and electrical stability of the hydrogel system by forming hydrogen bonds with the AM/CMC network. Subsequently, the solution was transferred to the second PDMS mold, which was exposed to UV light (365 nm) for 6 min to polymerize into a cross-linked ionic hydrogel with hierarchical microcones. A commercial AgNWs dispersion was used for the flexible electrodes. The AgNWs were dispersed in ethanol at a concentration of 0.01 mg mL^−1^. The typical diameter and length of AgNWs are 20–40 nm and 10–20 μm, respectively. Before use, the solution was stirred vigorously for 3 min until the nanowires were evenly dispersed in the solution. The AgNWs solution was then sprayed onto a 25 μm improved PVA substrate using an air spray gun and left to air dry until the solvent had completely evaporated, resulting in the formation of AgNWs-coated PVA electrodes. As illustrated in the cross section of the SEM image in Fig. S1, the ultrathin electrode effectively converts pressure into capacitance changes with high sensitivity. This improved PVA material remains stable at room temperature and is durable under daily human activities and exposure to sweat, exhibiting solubility only in hot water (above 65 °C), as shown in Fig. S2. Finally, the iontronic sensing device was assembled by sandwiching the ionic hydrogel with hierarchical microcones between two flexible AgNWs/PVA electrodes. This iontronic device demonstrated superior sensitivity compared to traditional sensors using elastomeric dielectrics such as PDMS [34].

### Design of Wireless Signal Acquisition Module and Software System

The wireless signal acquisition module contains a microcircuit board that includes a microcontroller, capacitance conversion chip, Wi-Fi wireless component (ESP-01S, see Table S1 for power consumption), and lithium polymer battery. Shielded wiring (AFPF, 0.035 mm^2^) was employed in our setup to minimize errors and prevent noise from radio frequency and electromagnetic interference (see Fig. S3 for the quantitative comparison of noise reduction). This compact module captures and measures wrist movements, transmitting them to the host computer at a rate of 12.5 Hz. To facilitate communication with the wireless module, a server based on the TCP/IP protocol was established on a computer for data transmission. Moreover, a graphical interface was developed using the PyQt library in Python to enhance the visualization of digital signals collected from microcontrollers. Three different applications for human–machine interaction were developed to suit various scenarios. Therefore, the computer continuously collects real-time capacitance signals and transmits them to the learning network for multitask recognition. Informed written consent was obtained from all human subjects before participation in the experiments.

### Time-Series Data Augmentation

In self-supervised contrastive learning, the design of diverse data augmentations plays a pivotal role in minimizing the distance between different views of the same sample while maximizing the distance from other samples [35, 36]. Traditional approaches for data augmentation in self-supervised contrastive learning typically create two random variants in the same direction from a sample *x*, resulting in two views, ${x}_{1}$ and ${x}_{2}$, using the same augmentation cluster $U$, ${x}_{1}\sim U$ and ${x}_{2}\sim U$. However, multiple experiments demonstrated that incorporating different data augmentation methods can enhance model representation robustness (Tables S2 and S3) [37]. Hence, we employed two distinct types of augmentations: strong and weak. Weak augmentations introduced limited variations to the original signal, such as time shifting and scaling. In contrast, strong augmentations introduce more significant perturbations in the shape of the signal while preserving its temporal trends. Upon conducting systematic research on various data augmentation methods (as presented in Table S2), we utilize scaling and jittering to generate weak augmentations, and jittering along with permutation to generate strong augmentations [38]. For each input of unlabeled data $x$, we define weak and strong augmentations as ${x}_{s}$ and ${x}_{w}$, respectively, where ${x}_{s}\sim {U}_{s}$ and ${x}_{w}\sim {U}_{w}$.

### Details of TS-VFC Learning

Our TS-VFC learning process comprises three main parts: encoder, cross-view fusion module, and projection head. For an input sample $x\in {R}^{4\times 32}$, where the signal contains 4 channels and the window size is set to 32, high-dimensional features are extracted using an encoder. We define $Z={f}_{enc}\left(x\right)$, $Z=\left[{z}_{1, }{z}_{2},\cdots {z}_{T}\right]$, where $T$ indicates the total timesteps, ${z}_{i}\in {R}^{d}$, and $d$ denotes the feature length. In this task, we set the timestep *T* = 9 and feature length *d* = 128, $\text{Z}\in {R}^{128\times 9}.$ Thus, we acquire two augmented views ${Z}^{s}$ and ${Z}^{w}$, which are the inputs of the fusion cross-view module.

We introduce a cross-view fusion method that facilitates semantic communication between views at different stages [39]. Ablation experiments (Table S3) show that the generalization ability and accuracy of the model can be improved through fusion between views at different stages. The specific method is as follows: in the total range of timestep $T$, ${T}_{r}$ steps are arbitrarily chosen to split the high-dimensional feature vector *Z* into two parts, and we define them as ${Z}_{k}=\{k|0\le k<{T}_{r}\}, { Z}_{g}=\{g|T-{T}_{r}\le g\le T\}$. ${V}_{k}$ is obtained using the Transformer model, where ${V}_{k}={f}_{tra}\left({Z}_{k}\right)$, which is used to fuse temporal view information ${Z}_{g}$. The strong augmentation produces ${V}_{k}^{s}$ and the weak augmentation produces ${V}_{k}^{w}$. For ${V}_{k}^{s}$ generated by strong augmentation, concatenation fusion is performed with ${Z}_{g}^{w}$ generated by the weak augmentation, and vice versa. The generated fused feature vectors are defined as ${V}_{k,g}^{s}$ and ${V}_{k,g}^{w}$. The Transformer is used for further feature extraction from the views to achieve cross-view fusion. It contains two main parts, multi-head attention and multiplayer perceptron block. We stack four identical layers to generate the regression features. Inspired by the BERT model, we include token $c$ as a representative vector in the input features of the model [40].

The projection head is responsible for mapping the views to the LTS to learn more discriminative representations. Assuming that $N$ input samples exist, two different augmentation methods provide two views for each sample, $2N$ in total. For ${V}_{k,g}^{i}$ from the cross-view fusion module and another augmented view ${V}_{k,g}^{{i}^{+}}$ from the same sample, we consider them as positive pairs, while the remaining $2N-2$ samples are treated as negative pairs. Therefore, the objective is to maximize the similarity between positive pairs and minimize the similarity between negative pairs. The loss function ${L}_{\text{TS-VFC}}$ is defined as:

1 $$L_{{\text{TS-VFC}}} = - \sum\limits_{{{\text{i}} = 1}}^{N} {\log \frac{{\exp (sim(V_{k,g}^{i} ,V_{k,g}^{{i^{ + } }} )/\tau )}}{{\sum\nolimits_{m = 1}^{2N} {{\mathbf{1}}_{[m \ne i]} \exp (sim(} V_{k,g}^{i} ,V_{k,g}^{m} )/\tau )}}} ,$$

where $sim\left(u,v\right)={u}^{T}v/\Vert u\Vert \Vert v\Vert$ denotes the cosine similarity between $u$ and $v$. $\tau$ symbolizes a temperature parameter and ${1}_{[m\ne i]}\in \left\{\text{0,1}\right\}$ represents an indicator function, only when $m\ne i$, ${1}_{[m\ne i]}$=1.

The loss function of fine-tuning stage: upon completing the pre-training of the model, few-shot labeled data for specific tasks were collected to fine-tune the model. The cross-entropy loss function ${L}_{\text{fine}}$ is expressed as

2 $$L_{{\text{fine}}} = - \sum\nolimits_{i = 1}^{N} {y_{i} \log (\hat{y}_{i} )} .$$

### Code and Data Availability

The source code used for TS-VFC Learning and source data in this study are available at https://drive.google.com/drive/folders/18fM5DNxor0Ahy7CXhHWjc5MGnI2cIYrv?usp=sharing.

## Results and Discussion

### Sensing Mechanism and Properties of Iontronic Device

**Fig. 2:**
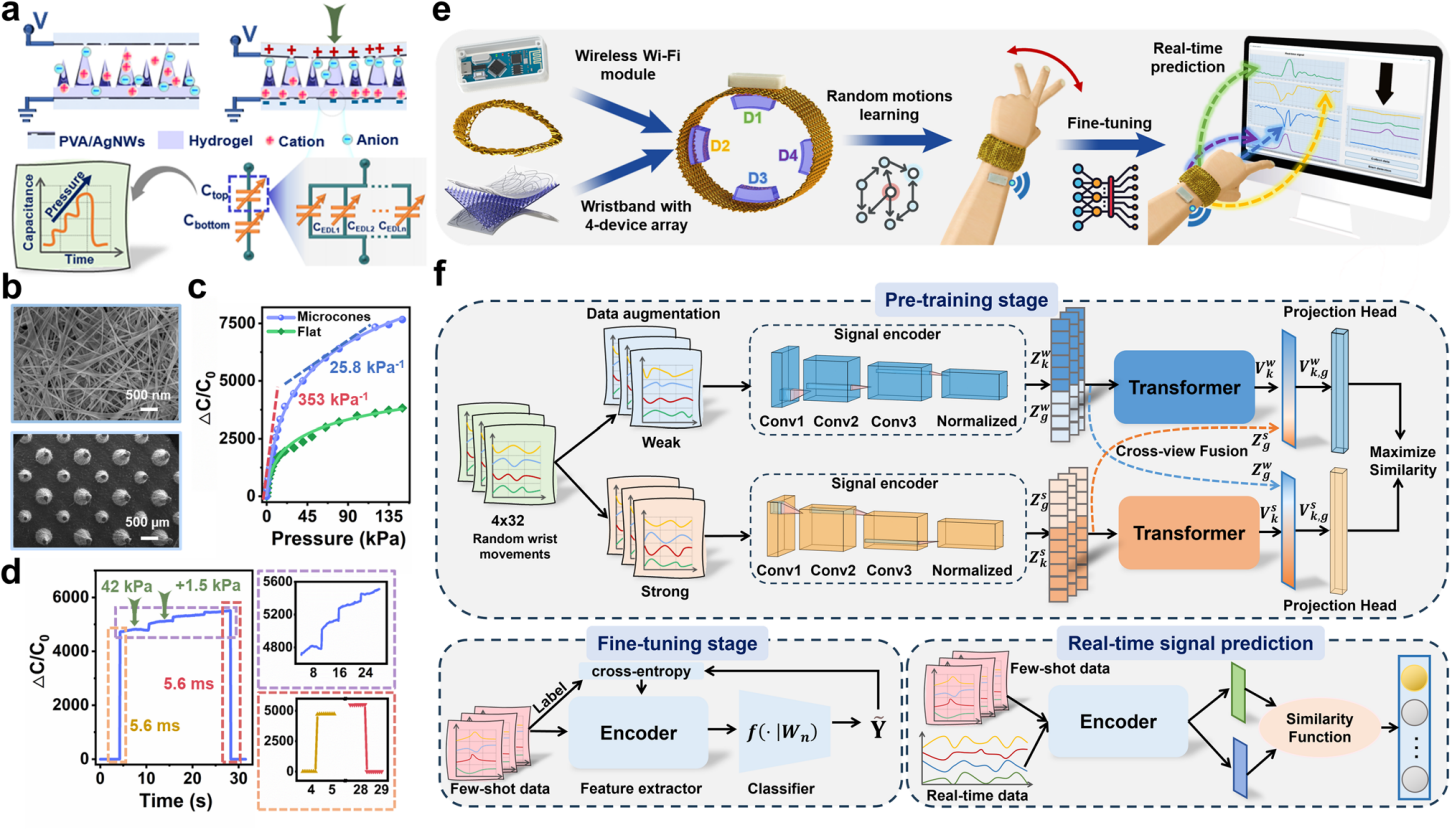
Sensing mechanism and properties, and TS-VFC algorithm architecture. **a** Schematic of the sensing mechanism of iontronic device. **b** Scanning electron microscope (SEM) images of ionic hydrogel with microcone structure and AgNWs/PVA electrode. **c** Normalized change of capacitance as a function of pressure over 150 kPa. **d** Response of tiny pressure (1.5 kPa) variations when high pressure (42 kPa) has been preloaded. Response and recovery time of the device (5.6 and 5.6 ms). **e** Illustration of general prediction process, including preparation of wristband with attached sensing devices and wireless module, TS-VFC learning of random motions, few-shot fine-tuning, and real-time signal prediction. **f** Details of TS-VFC learning model architecture. During the pre-training step, the overall architecture of the proposed TS-VFC model performs contrastive training through unlabeled random data. The encoder is then fine-tuned through supervised learning given few-shot labeled data. Finally, the encoder is used to encode real-time signals, metric distance from encoded label data, and complete signal category prediction

The detailed illustration of the pressure-sensing mechanism of the iontronic device is presented in Fig. 2a. The photocurable hydrogel employed as the dielectric layer in the device plays a critical role in sensing. When voltage is applied across the AgNWs electrodes (Fig. 2b), cations and anions within the hydrogel migrate and gather at interfaces between electrodes and hydrogel, creating an electric double layer (EDL) [41, 42]. The crucial characteristic of this layer is its nanometer-scale thickness, resulting in extremely high capacitance. Hierarchical microcones are incorporated within the dielectric hydrogel layer to enhance the contact area variation under pressure [43, 44]. Figures 2b and S1 display hierarchical microcones with two different heights (680 and 500 μm), diameters (450 and 350 μm), and a spacing of 1200 μm. As demonstrated in the finite element simulation in Fig. S4, the low pressure primarily influences the contact area variation of the higher microcones. As the pressure increases, smaller microcones sequentially contact the upper electrode after the compression of the taller ones. The resulting high interfacial capacitors (C_EDL1_, C_EDL2_…C_EDLn_) are crucial for achieving high sensitivity because they undergo significant changes with variations in the contact area owing to the pressure-induced deformation of the microcones.

Sensitivity, mathematically expressed as *S* = ∂(Δ*C*/*C*_0_)/∂*P*, *C*_0_ represents the initial capacitance without any applied pressure, Δ*C* expresses the variation in capacitance after applying pressure, and *P* denotes the applied pressure. Figure 2c illustrates the capacitance response curves of the iontronic devices, both with and without hierarchical microcones. In the case of the device featuring the microcones, sensitivity reaches 353 kPa^−1^ within the lower-pressure range and 25.8 kPa^−1^ in the higher-pressure range, outperforming the device with a flat hydrogel layer across the entire pressure range. Owing to its high sensitivity and broad sensing range (150 kPa), the device maintains a high pressure resolution throughout the pressure range. Thus, even under a preloaded high pressure of 42 kPa, the device can discern slight pressure variations (1.5 kPa), as depicted in Fig. 2d. Furthermore, it shows a fast response and recovery times of 5.6 ms during both loading and unloading pressures. Furthermore, as illustrated in Fig. S5, the device exhibits excellent stability throughout the repeated loading and unloading processes at different pressures (2.5, 8, 13, 22, 43, and 80 kPa). No significant differences in capacitance values were observed at the same pressure levels. Furthermore, the device exhibits stable and synchronous response during repeated bending at different angles (30°, 45°, 60°, and 80°), as shown in Fig. S6. To assess device durability, a cyclic pressure of 30 kPa was applied over 15,000 cycles, and the capacitive response is shown in Fig. S7. The inset displays the waveforms from the last few cycles of the device, which remained nearly unchanged throughout the test. Additionally, Fig. S8 illustrates that the output signal essentially maintains the same level after 11,000 cycles of bending at 65°, ensuring long-term use in daily activities.

### Rapid Adaptation of Multi-Scenario Tasks by Contrast Learning

Previous studies on wrist recognition models were limited by their specificity to individual tasks, lacking the adaptability to handle multiple scenarios [22, 23]. We aim to develop a robust and general model that can rapidly adapt to wrist movements in unfamiliar scenarios for daily tasks. Figure 2e depicts a block diagram that illustrates the entire prediction process. The wristband contains four iontronic devices worn on the wrist, complemented by an attached wireless Wi-Fi module to ensure user comfort and create a self-contained system (Fig. S9 and Movie S1). Instead of using labels to classify the four capacitance signals, the proposed model employs unlabeled random wrist-motion signals for self-supervised contrastive learning, distinguishing it from traditional supervised algorithms. Using this approach, features from the sensor signals are autonomously learned, resulting in a separable feature space that enables classification for different tasks. The fundamental principle involves calculating the similarity between the identical and different samples, creating the LTS with a strong representation of the signals generated by the smart wrist to represent prior index finger movements. Consequently, new users can rapidly engage in transfer learning for various tasks, facilitating the division of distinct samples. When undertaking a new task, only few-shot of wrist-motion data specific to that task need to be collected. These signals are then projected onto the LTS. Through metric calculations, primarily based on cosine similarity, and comparisons of these features with real-time inputs, precise predictions of wrist motion gestures can be displayed on the screen, even for tasks not included in the training set of the model.

To achieve rapid adaption to wrist motions across multiple scenarios, we propose a TS-VFC algorithm trained with a large amount of unlabeled time-series data. Figure 2f illustrates the detailed architecture and workflow of the proposed algorithm, which contains three distinct stages. In the pre-trained stage, unique wrist movements and postures, generated by new users wearing the wristband, result in randomly changing signals from the devices. These unlabeled signals are utilized to create a general LTS through the TS-VFC learning process. First, data augmentation methods are employed to generate two correlated views for each time series. These views are referred to as positive pairs as they originate from the same wrist-motion signal, while sequences with different actions serve as negative pairs. A variety of data augmentation methods, including both ‘weak’ and ‘strong’ approaches, are used to produce these correlated views (Refer to the Experimental Section for details on data augmentation methods). Subsequently, an encoder, comprising three one-dimensional convolutional layers (Table S4), is utilized to extract high-dimensional latent features from the generated views [45]. A cross-view fusion module is introduced to calculate the time-series contrast loss by merging views at different stages. The transformer architecture (Fig. S10) serves as the implementation of this module owing to its robust feature extraction capabilities and high efficiency [46]. A neural network projection head composed of two linear layers is employed to map feature vectors to the LTS [47]. Finally, the similarity function minimizes the distance between pairs of positive samples in the LTS, thereby training the potent feature encoder that can be applied to various downstream tasks.

In the fine-tuning stage, building upon the pre-trained model weights, we collected few-shot label data and introduced a traditional supervised learning model to fine-tune the LTS. The fine-tuning process is essential for achieving transfer learning, ensuring that the LTS can effectively handle complex wrist movements across different scenarios. To accomplish this, a classifier in the form of a linear layer was integrated into the last layer of the encoder. This supervised training step facilitates the adaptation of the model to the specific task at hand. Recognizing the diversity in user writing habits within the same scenario, which can manifest as variations in writing time, intensity, and pause time for each stroke, it is essential to normalize these writing habits. The normalization process helps minimize signal deviations within the same action, as illustrated in Figs. S11 and S12. For real-time signal prediction, the encoder is used to map the signals generated by the real-time wrist movements of the user into high-dimensional features. These features are then compared with the prior movements already embedded in the LTS through metric calculations, enabling the prediction of wrist-motion categories [48, 49].

### Prediction of Four/Eight Directions and Game Control

**Fig. 3:**
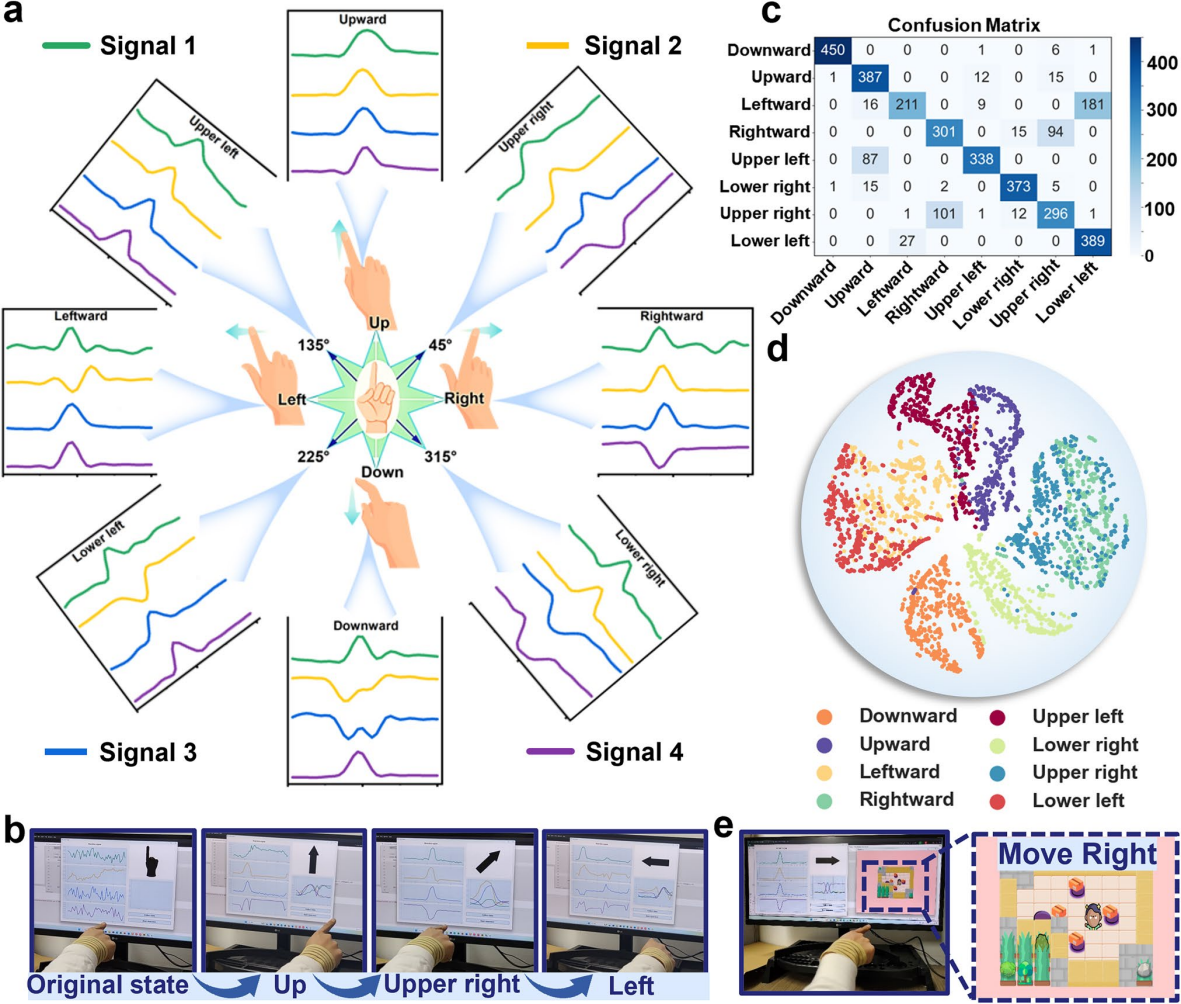
Demonstration of direction prediction and game control. **a** Waveforms of real-time four-channel signals of eight directions. **b** Photos of real-time prediction for direction recognition with the wristband. **c** Confusion matrix for prediction of eight different directions, with an average accuracy of 82.0%. **d** t-SNE projection of high-dimensional latent features of labeled data for eight-direction recognition using transfer learning. **e** Demonstration of game control and screenshots of the game interface

When the user points their index finger in different directions with the wristband, the corresponding capacitance signals from the four devices are captured from the wireless module. Real-time direction predictions can be achieved by fine-tuning the labeled motion data specific to directions. Figure 3a illustrates the corresponding signal waveforms when the index finger moves in eight different directions. Moving the index finger horizontally to the right represents a right command (0°), and moving it diagonally upward to the right by 45° indicates an upper right command (45°). Notably, discernible differences exist in the time-varying outputs of Signal 2, even for similar gestures (such as left and lower left). The proposed model is designed to effectively adapt to new actions introduced into a few-shot labeled dataset while preserving the information from the original actions. The training process does not need to be strengthened, and the model does not need to be redesigned for newly added actions. While initially trained to recognize four basic directions (left, right, up, and down), the proposed model can effortlessly expand its capabilities to recognize four additional directions (45°, 135°, 225°, and 315°) by simply supplementing the corresponding few-shot labels. Images of real-time predictions are illustrated in Fig. 3b, demonstrating continuous changes in the four-channel signals and corresponding predicted results on the interface. The entire recognition process for both four and eight directions, encompassing prior motion learning, is demonstrated in Movies S2 and S3, respectively. The distinction is that for the four directions, only 3-shot data are required in each direction for a total of 12 actions for transfer learning. Conversely, for the eight directions, only 5-shot data are required in each direction to complete the fine-tuning.

To validate the performance of the model, confusion matrices were generated for recognizing either four or eight directions in the test set after 80 transfer training epochs. These matrices indicate that the proposed model achieved average accuracies of 94.7% for the four directions (Fig. S13) and 82.0% (Fig. 3c) for the eight directions, respectively. To improve the accuracy of predicting eight directions, adding more labeled data (e.g., from 5-shot to 10-shot) during the fine-tuning phase is effective. As shown in Fig. S14, the accuracy of predicting the eight directions reached 87.5% with 10-shot. Our proposed algorithm allows users to customize the number of training samples, enabling adjustments based on personal needs. For example, users can choose a smaller number of labeled data for rapid adaptation or a larger number of labeled data to improve accuracy. We employed the t-distributed stochastic neighbor embedding (t-SNE) algorithm to perform dimensionality reduction on the high-dimensional features extracted from the encoder to provide further insight and analysis of the recognition of these actions (Figs. 3d and S15) [50]. In this feature space, the feature points of each category are accurately mapped to their corresponding positions for these directions, underscoring the robustness of the proposed model in this scenario. To demonstrate the practical interaction capabilities of the wristband, we utilize direction recognition for controlling a Sokoban game. As indicated in Fig. 3e, the capacitive outputs generated by the index finger motions are captured and transmitted by the wireless module, subsequently read by a computer program to predict the direction. This prediction is then used to control the movement direction of the game character in a customized game (Movie S4).

### Prediction of Air-Writing Numbers and Letters

**Fig. 4:**
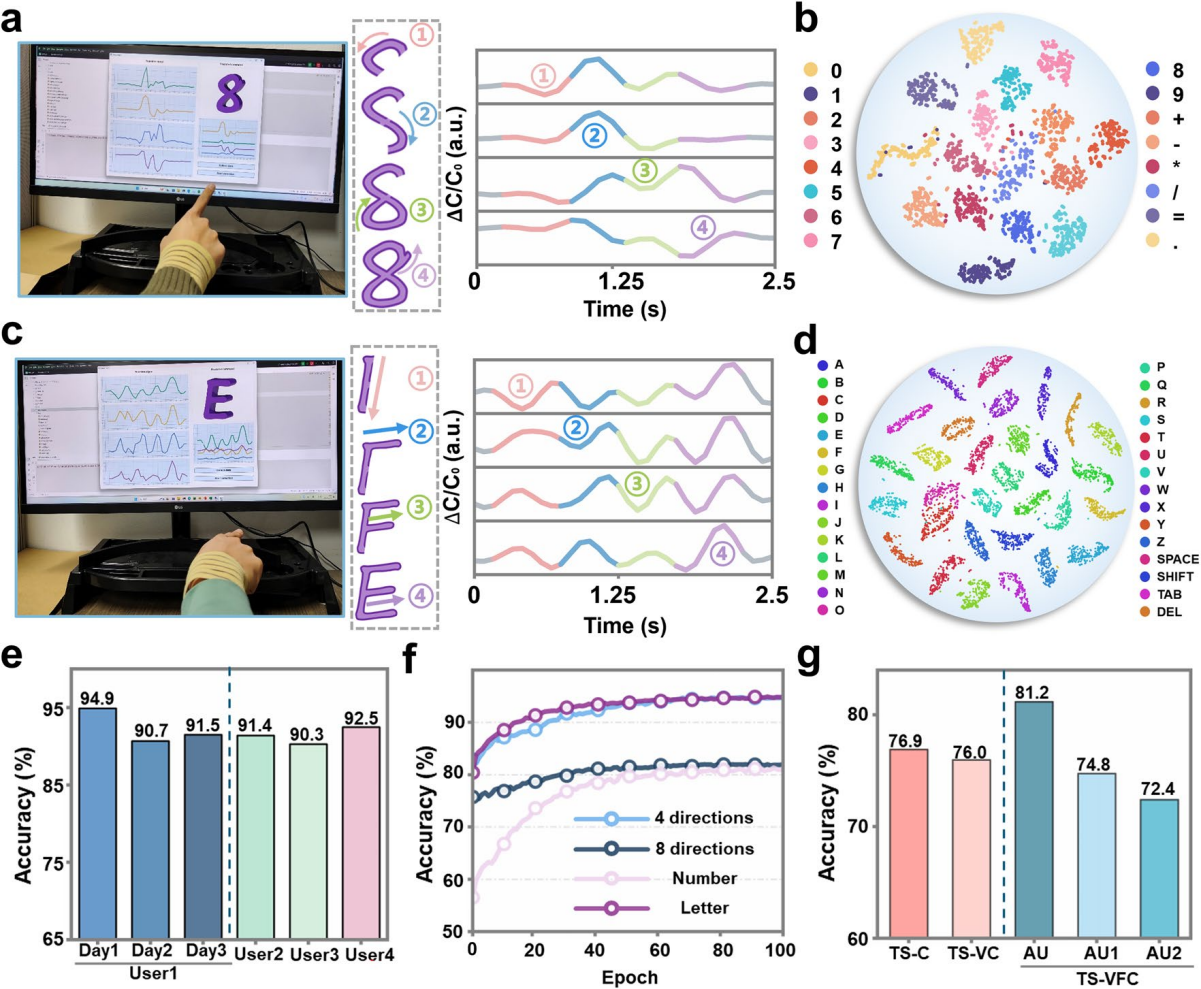
Demonstration of air-writing numbers and letters. **a** Photo of real-time number prediction, as well as detailed process and waveform of air-writing “8”. **b** t-SNE projection of high-dimensional latent features for the test set using transfer learning. **c** Photo of real-time letter prediction, as well as detailed process and waveform of air-writing “E”. **d** t-SNE projection of high-dimensional features for test set of 26 letters and four function keys. **e** Comparison of accuracy in air-writing 26 letters and 4 function keys by the same user at different times and by 4 different users. **f** Accuracy trends for different tasks during fine-tuning training epochs: under the weights of a pre-trained model, supervised training is conducted with few-shot labeled data by adding a linear layer behind the encoder model. **g** Ablation experiment study of different variants of the TS-VFC model and using different data augmentation methods for number prediction. TS-C model without view fusion and Transformer architecture, and TS-VC model with the Transformer architecture. AU (TS-VFC model) uses “scale + jitter” for weak augmentation and “jitter + permutation” for strong augmentation. AU1 uses “scale + jitter” for both weak and strong augmentation, and AU2 uses “jitter + permutation” for both weak and strong augmentation

Based on the excellent direction recognition ability of the wristband, we extend its functionality to handle more complex tasks, such as recognition of numbers and letters, by analyzing the features of the signals generated when the index finger moves in different directions. Figure 4a illustrates the scenario of air-writing numbers with the wristband, along with the interface displaying the four-channel signal and prediction. The number “8” is identified by extracting the capacitance signals during the writing process. The consecutive writing process is divided into four steps (①–④), with each sensor producing corresponding signals that exhibit continuous but different waveforms. In addition, mathematical operators (such as +, −, =) are included in the learning scope to develop the calculator system, and their waveforms differ from those of the numbers, following the writing format, as illustrated in Fig. S11. To standardize the writing methods, the finger returns to its initial position after completing the last stroke of each character. The four-channel signal diagram for all numbers “0–9” and six operators are presented in Fig. S16. The proposed model achieves an average accuracy of 81.2% in predicting 16 categories of numbers and symbols with only 5-shot learning for each category (Fig. S17), eliminating the necessity for a large volume of labeled data for training. As illustrated in Fig. 4b, employing the t-SNE algorithm to visualize these features and project them onto the feature space reveals that each category forms distinct clusters.

Similar to the number prediction approach described earlier, the wristband can recognize all 26 English letters through air-writing pattern tracking. Considering the letter “E” as an example, the waveform characteristics of the corresponding signal are analyzed. Figure 4c demonstrates that it takes four strokes to write the letter “E,” specifically down-right-right-right (①–④), with the finger returning to the initial position after the last stroke. Since the strokes from ② to ④ are identical, it is evident that the separated signal features are also similar. Furthermore, four function keys were added to develop the keyboard input system encompassing 26 letters and four function keys. The specific writing methods for these function keys are defined in Fig. S12. The four-channel signal diagram for all letters “A–Z” and four functional keys is presented in Fig. S18. Owing to the increased complexity of these actions and the consequently greater semantic information collected from the four-channel signals, the proposed model achieves an average accuracy of 94.9% for letter and function key recognition with only 5-shot learning (Fig. S19). Figure 4d displays the test set containing these 30 categories, extracted using the proposed model, and projected into the latent space using the t-SNE algorithm for dimensionality reduction. A significant distance exists between each category within this space, enabling character recognition within this scenario by calculating the distances between each category.

Movie S5 showcases the steady and continuous recognition of the letters “K B G I F M V P.” When the same user writes these letters at different times, as shown in Fig. S20, the four-channel signal changes are only slightly different. While prolonged wear may lead to sliding between the wristband and skin, which could affect device output and recognition precision, the simultaneous operation of the four sensing devices and TS-VFC learning algorithms ensures consistent performance even when slight positional shifts of the wristband occur over time (Figs. 4e, S21 and S22). Despite potential variations among individuals, the proposed model can consistently learn the writing characteristics of new users. When four users with different wrist sizes write these eight letters, the changes in the four-channel signals are illustrated in Fig. S23. As shown in Fig. S24, the wrist of the overweight male (user 4) is relatively thick, with a circumference greater than 20 cm, while the slender female (user 3) has a thinner wrist with a circumference less than 15 cm. The wrist sizes of users 1 and 2 are average, approximately 17.5 cm. Although these signals may be different, new users only need to provide few-shot labeled data to achieve the desired prediction effect (Figs. 4e and S25–S27). Considering that some symbols have the same strokes but are arranged in different relative positions, to demonstrate that the wristband is capable of restoring, reproducing, and reflecting the spatial trajectory of the strokes rather than just recognizing the strokes, we designed additional air-writing symbol experiments. The writing sequence of the symbols with the same stroke features and their corresponding four-channel signals are shown in Fig. S28. Through our TS-VFC learning with 5-shot, the prediction accuracy can reach 88.6% (Fig. S29). Given the widespread use of commercial accelerometers for motion tracking, a comparison was conducted to evaluate the performance of our proposed device. We collected signals from air-writing letters A to H using the accelerometer, as shown in Fig. S30. The similarity matrices for the signals corresponding to these 8 letters from both sensors were calculated (Fig. S31), and the accuracy of predictions was trained using TS-VFC learning under the same conditions (Fig. S32). The results demonstrate that the flexible sensing device provides superior performance for continuous and accurate monitoring of subtle wrist movements during air-writing applications.

Figure 4f illustrates the capability of the pre-trained model learned by the TS-VFC algorithm to adapt to different scenarios. Without any fine-tuning training (epoch = 0), the pre-trained model already initially adapts, and it can achieve over 80% accuracy within the transfer training period of 40 epochs. Following 80 epochs of fine-tuning training, the accuracy and MF1-score results that correspond to each scenario are presented in Table S5. Our pre-trained TS-VFC model demonstrates robust prediction capabilities across multiple scenarios. In addition, taking the number prediction scenario as an example, we perform ablation experiments by constructing different model variants to compare with the TS-VFC model, aiming to demonstrate the effectiveness of each component. The TS-C module lacks both view fusion and Transformer architecture, while the TS-VC module incorporates the Transformer architecture. As illustrated in Fig. 4g, the experiment results reveal that the proposed TS-VFC model improves accuracy by over 4% for these two variants. This is attributed to the use of the Transformer to achieve view fusion at different stages, which helps positive samples from the same class generate more discriminative features. The comparison of ablation experiments for other tasks, including direction and letter prediction, is provided in Table S3. Subsequently, we explore the effects of weak and strong data augmentation on the TS-VFC model. As indicated in AU1 and AU2 (Fig. 4g), when only two weak or two strong augmentations are used, the accuracy falls by approximately 9% compared to the proposed TS-VFC model. Evidently, merely using weak augmentation (AU1) fails to learn more discriminative features owing to minor data variations. Conversely, relying solely on strong augmentation (AU2) prevents the model from recognizing the original signal during prediction. To validate the rationality of the proposed data augmentation method selection, we conducted a detailed study on the impact of various augmentation techniques on the TS-VFC model in number and letter prediction, as shown in Table S2.

### Demonstration of Air-Writing Input System

**Fig. 5:**
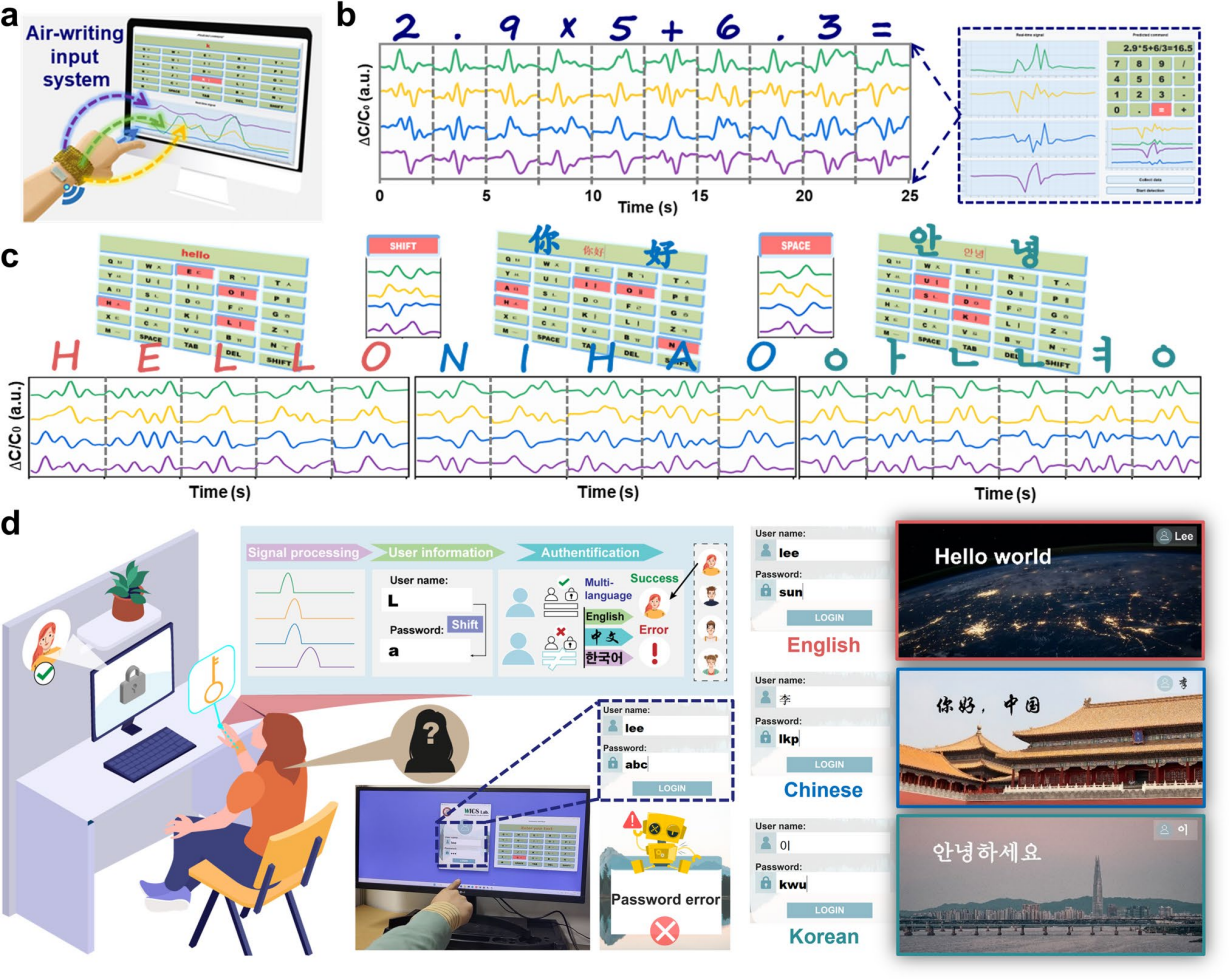
Illustration of the calculator, keyboard input, and login system based on air-writing. **a** Schematic of air-writing input system. **b** Waveforms of four signals during the calculation operation process, and a screenshot of the calculator interface. **c** Screenshots of keyboard input interface, and waveforms of four signals of air-writing words “hello”, “你好” and “안녕”. **d** Schematic of multi-language login system based on the wristband. Real-time prediction of inputting username and password, and screenshots of login interface

For the air-writing input system developed based on the wristband (Fig. 5a), signals from the wristband are transmitted to the computer through the wireless module for further processing and recognition, with the prediction results displayed on the interface in real time. The calculator system serves as a ubiquitous calculation tool in our daily lives, and Fig. 5b presents the continuous variations in the four-channel signals while air-writing the equation “2.9 × 5 + 6/3 =”. The corresponding characters were identified in real time, and the final result of the Eq. (16.5) can be obtained after entering the symbol “=”. Movie S6 depicts the operational process of the calculator, demonstrating rapid and stable distinguishing between different numbers and symbols by air-writing with an index finger.

As depicted in Fig. 5c, the signal variations when air-writing greetings in three languages (English, Chinese, Korean), are switched using the function key “SHIFT.” The word “HELLO” is initially air-written with the wristband, and waveforms of responses are displayed on screen in real time, with corresponding words recognized. The “SHIFT” and “SPACE” functions are represented by specific strokes (Fig. S12), and their waveforms differ from those of the other letters. Upon switching the input method, “你好” and “안녕” continue to be entered and are successfully recognized, as demonstrated in Movie S7. A further developed login system that supports multi-language input is illustrated in Fig. 5d, and users can access various language systems by switching their handwritten username inputs. In the scenario where the user enters the password “abc” corresponding to the username “lee” in the login system, which does not match the preset correct password “sun,” the interface will automatically prompt “Password error.” Upon entering the correct username and password, users can freely switch to the login system in different languages following successful authentication. Each interface is shown in Fig. 5d, and the overall process of handwritten input, password verification, and interface display is shown in Movie S8. The capability to convert air-written characters into signals comprehensible to computers, without the need for learning proprietary touch symbols, minimizes user burden, and it is particularly useful for individuals with visual impairments.

## Conclusions

Leveraging the innovative capabilities of TS-VFC learning, we introduced a wearable wristband designed to conform to wrist anatomy (see Fig. S33 for the breathability experiment of the wristband material), facilitating rapid adaptation to diverse scenarios of dynamic gesture tracking. As indicated in Table S6, compared with other representative studies of wristband systems, our innovative approach enables transfer learning utilizing unlabeled data. Each iontronic sensing device within the wristband, featuring hierarchical microcones and ultrathin flexible electrodes, has demonstrated exceptional performance, boasting a high sensitivity of 353 kPa^−1^ and a board response range of 150 kPa. Moreover, we designed a compact circuit with a Wi-Fi module for the wireless acquisition of four-channel signals from the array. Following the formation of the LTS during the pre-training stage in the model, the wristband system has proven effective in handling various tasks across multiple scenarios. These include the prediction of precise eight-direction commands and air-writing of numbers and letters. Despite the encoder in the model comprising only three layers of one-dimensional convolutions, it can adapt to new gesture recognition tasks without requiring architectural redesigning or extensive training for specific tasks. With minimal labeled data, the model can be fine-tuned for generalization to new tasks, even those not originally included in the training data. Moreover, practical applications, such as game control, calculator operation, and login systems, were demonstrated, highlighting the feasibility and potential of human–machine interaction. This self-supervised wristband system seamlessly integrates with a user, offering an intuitive means of communication and control of digital interfaces through gestures that align with everyday habits. It has the potential to provide a more natural, immersive, efficient, and personalized digital experience in the future.

## Supplementary Information

Below is the link to the electronic supplementary material.

Supplementary file1 (MP4 6167 kb)

Supplementary file2 (MP4 11016 kb)

Supplementary file3 (MP4 24967 kb)

Supplementary file4 (MP4 4670 kb)

Supplementary file5 (MP4 8799 kb)

Supplementary file6 (MP4 7158 kb)

Supplementary file7 (MP4 16211 kb)

Supplementary file8 (MP4 20795 kb)

Supplementary file9 (DOCX 23699 kb)
