# Supplementary material for: A Rapid Adaptation Approach for Dynamic Air-Writing Recognition Using Wearable Wristbands with Self-Supervised Contrastive Learning
Source: Nanomicro Lett. 2024 Oct 16;17:41. doi: 10.1007/s40820-024-01545-8 (PMC11480301; doi:10.1007/s40820-024-01545-8)
Supplement: Supplementary file 9 — Supplementary file9 (DOCX 23699 kb) [file 40820_2024_1545_MOESM9_ESM.docx]

Supporting Information for

**A Rapid Adaptation Approach for Dynamic Air-Writing Recognition Using Wearable Wristbands with Self-Supervised Contrastive Learning**

Yunjian Guo^1,#^, Kunpeng Li^1,#^, Wei Yue^2,3,#^, Nam-Young Kim^2,3^, Yang Li^4,5,^*, Guozhen Shen^6,^* and Jong-Chul Lee^1,^*

^1^ Department of Electronic Convergence Engineering, Kwangwoon University, Seoul 01897, South Korea

^2^ Radio Frequency Integrated Circuit (RFIC) Bio Centre, Kwangwoon University, Seoul 01897, South Korea

^3^ Department of Electronic Engineering, Kwangwoon University, Seoul 01897, South Korea

^4^ School of Microelectronics, Shandong University, Jinan 250101, P. R. China

^5^ State Key Laboratory of Integrated Chips and Systems, Fudan University, Shanghai, 200433, P. R. China

^6^ School of Integrated Circuits and Electronics, Beijing Institute of Technology, Beijing 100081, P. R. China

^#^Yunjian Guo, Kunpeng Li and Wei Yue contributed equally to this article.

*Corresponding authors. E-mail: [yang.li@sdu.edu.cn](mailto:yang.li@sdu.edu.cn) (Yang Li); [gzshen@bit.edu.cn](mailto:gzshen@bit.edu.cn) (Guozhen Shen); [jclee@kw.ac.kr](mailto:jclee@kw.ac.kr) (Jong-Chul Lee)

**S1 Experimental Section**

**S1.1 Data Preprocessing Method**

The proposed model relies on three types of datasets: a pre-trained unlabeled dataset, a few-shot labeled dataset specific to a scenario, and a test set validated for that scenario. The unlabeled dataset consisted of randomly generated wrist-motion signals from three users, totaling 2,000 s of random wrist-motion signals, which were used to pretrain the model. We selected three different scenarios (directions, numbers, and letters) and gathered few-shot labeled data (five shots per scenario) for fine-tuning. To effectively assess the representation capability of our learning framework under constrained conditions, we utilized a limited labeled dataset instead of pre-trained weights for initialization during the training process. For instance, the wrist starts in a still position, and then numbers or letters are air-written with the index finger. The first complete movement after returning to the initial state following writing was considered a collection cycle. To accurately reflect the real usage scenarios of new users, the test set was generated through users’ normal interactions with the software interface.

Given that different individuals may exhibit differences in baseline capacitance values while wearing the wristband, the four-channel signals needed to be normalized using a method based on maximum and minimum values:

$X_{i}=\frac{C_{i}-\min(C_{i})}{\max(C_{i})-\min(C_{i})},\text{ }i=1,2,3,4$ (S1)

The pre-trained unlabeled datasets and few-shot labeled datasets were normalized separately. For the test set, the normalized maximum and minimum values were obtained from the labeled dataset specific to that scenario. For any set of collected capacitance signals (comprising four channels) $C=\{C_{1},C_{2},C_{3},C_{4}\}, C_{i}=[c_{i,1},c_{i,2},\cdots,c_{i,j}]$, where $j$ indicates the signal length and the normalized signal for each channel is represented as $X$. The original capacitance signals were susceptible to noise interference. Low-pass filtering techniques were applied to the data signals to eliminate unnecessary noise and minimize signal amplitude distortion effects (see Fig. S3 for the quantitative comparison of noise reduction).

**S2** **Supplementary Figures**


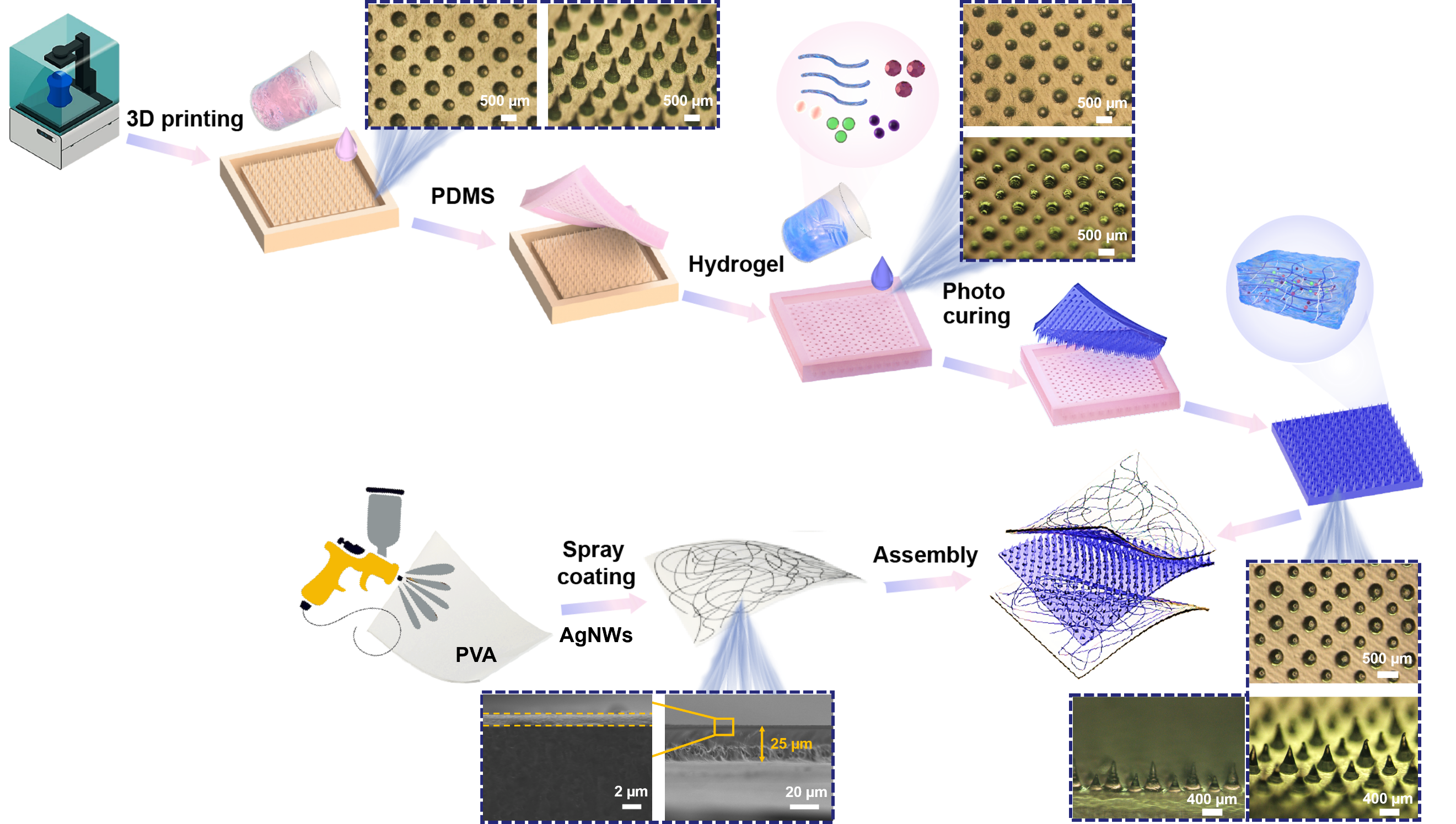


**Fig. S1** Schematic of detailed flowchart of iontronic sensing device and microstructure images corresponding to each stage


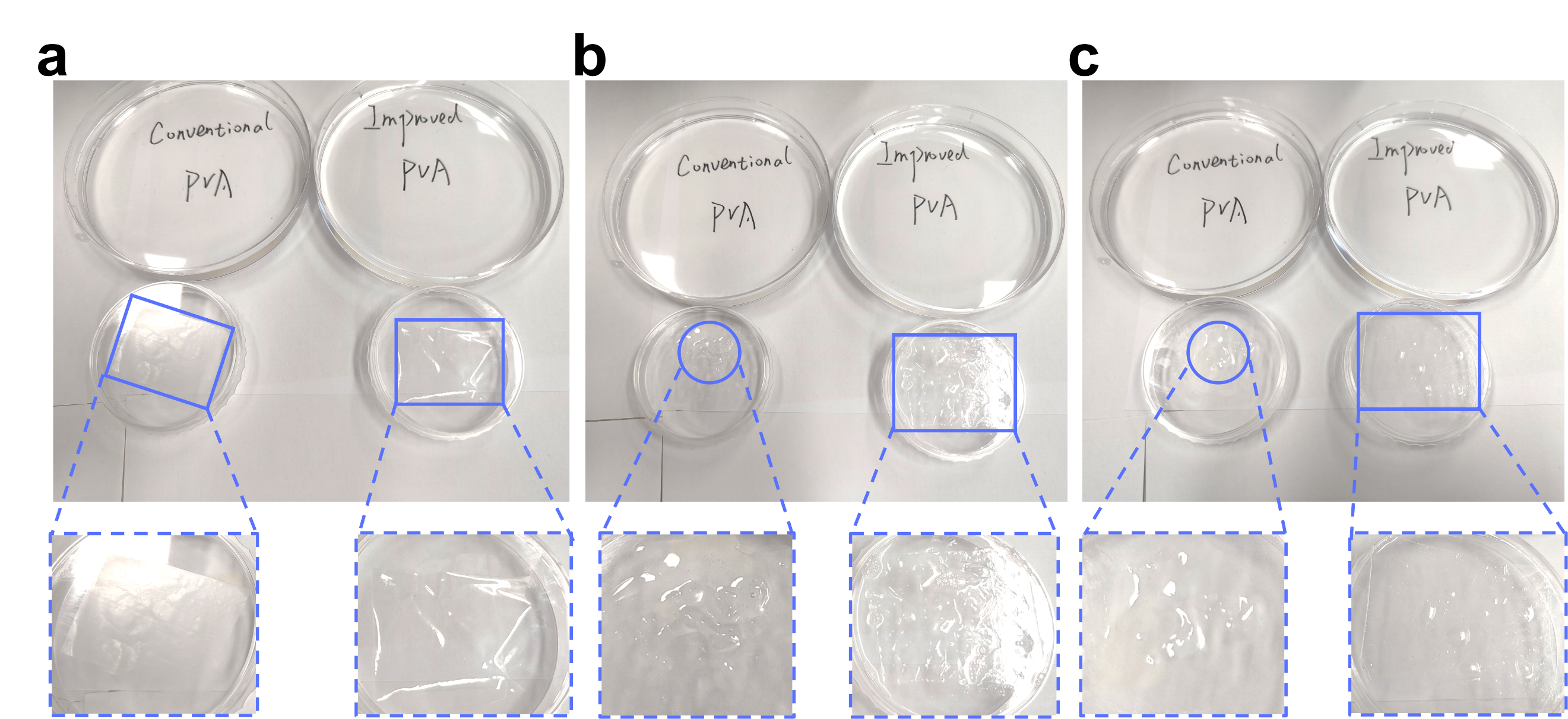


**Fig. S2** Comparison of the initial states of conventional PVA and improved PVA, their states after dissolving in water at room temperature (28°C) for 60 s, and their states after drying for 200 s


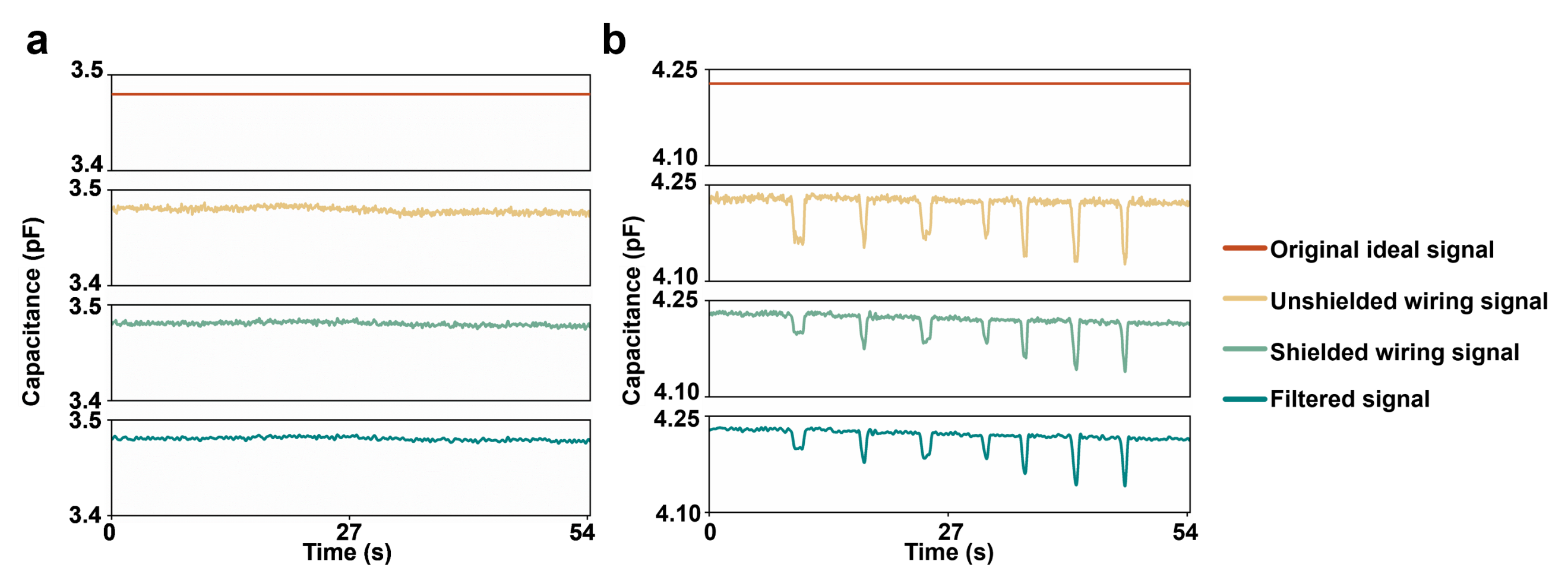


**Fig S3** Waveform diagrams of four signals: **a** in the pressure-free static state and **b** under low pressure with external metal interference

To demonstrate that shielded wiring and low-pass filtering effectively reduce noise in capacitive sensors, the signal-to-noise ratio (SNR) is calculated for quantitative data comparison and analysis. Assuming that the original ideal signal is A=[*a*_1_, *a*_2_,…*a*_n_], the signal with unshielded wiring is B=[*b*_1_, *b*_2_,…*b*_n_], the signal with shielded wiring (before low-pass filtering) is C=[*c*_1_, *c*_2_,…*c*_n_], and the signal after low-pass filtering is D=[*d*_1_, *d*_2_,…*d*_n_].

The SNR of unshielded wiring signal is *SNR_unshielded_*:

$$SNR_{\text{unshielded}}=10*\log_{10}\left(\frac{\frac{1}{N}\sum_{i=1}^{N} \text{a}_{i}^{2}}{\frac{1}{N}\sum_{i=1}^{N} \left(b_{i}-a_{i}\right)^{2}}\right)$$

The SNR of shielded wiring signal is *SNR_shielded_*:

$$SNR_{\text{shielded}}=10*\log_{10}\left(\frac{\frac{1}{N}\sum_{i=1}^{N} \text{a}_{i}^{2}}{\frac{1}{N}\sum_{i=1}^{N} \left(c_{i}-\text{a}_{i}\right)^{2}}\right)$$

The SNR of signal before filtering is *SNR_before_*:

$$SNR_{\text{before}}=SNR_{\text{shielded}}=10*\log_{10}\left(\frac{\frac{1}{N}\sum_{i=1}^{N} \text{a}_{i}^{2}}{\frac{1}{N}\sum_{i=1}^{N} \left(c_{i}-\text{a}_{i}\right)^{2}}\right)$$

The SNR of signal after filtering is *SNR_after_*:

$$SNR_{\text{after}}=10*\log_{10}\left(\frac{\frac{1}{N}\sum_{i=1}^{N} \text{a}_{i}^{2}}{\frac{1}{N}\sum_{i=1}^{N} \left(\text{d}_{i}-\text{a}_{i}\right)^{2}}\right)$$

The differences in these four SNR values for two measured signals were analyzed separately, as shown in Fig. S3. For the first signal, measured in a stationary state without pressure (Fig. S3a), the values are: ***SNR_unshielded_* = 58.8**, ***SNR_shielded_* = *SNR_before_* = 61.5**, **and *SNR_after_* = 62.1**. For the second signal, measured under low pressure with external metal interference (Fig. S3b), the values are: ***SNR_unshielded_* = 45.9**, ***SNR_shielded_* = *SNR_before_* = 47.7**, **and *SNR_after_* = 47.9**. These results show that both shielded wiring and low-pass filtering improve the SNR of the capacitive signal, effectively reducing noise in the collected data.

**
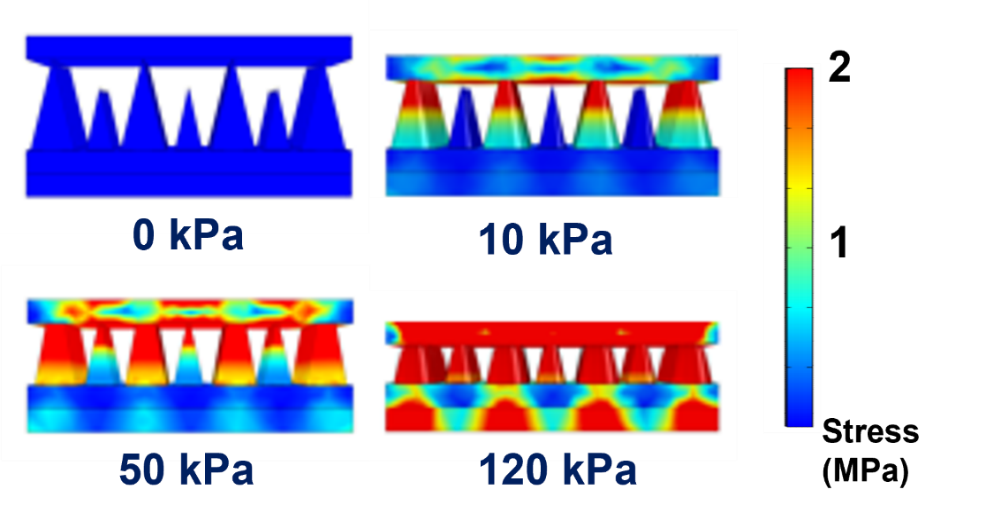
**

**Fig. S4** Finite element analysis result of the stress distribution of device with hierarchical microcones under pressures up to 120 kPa


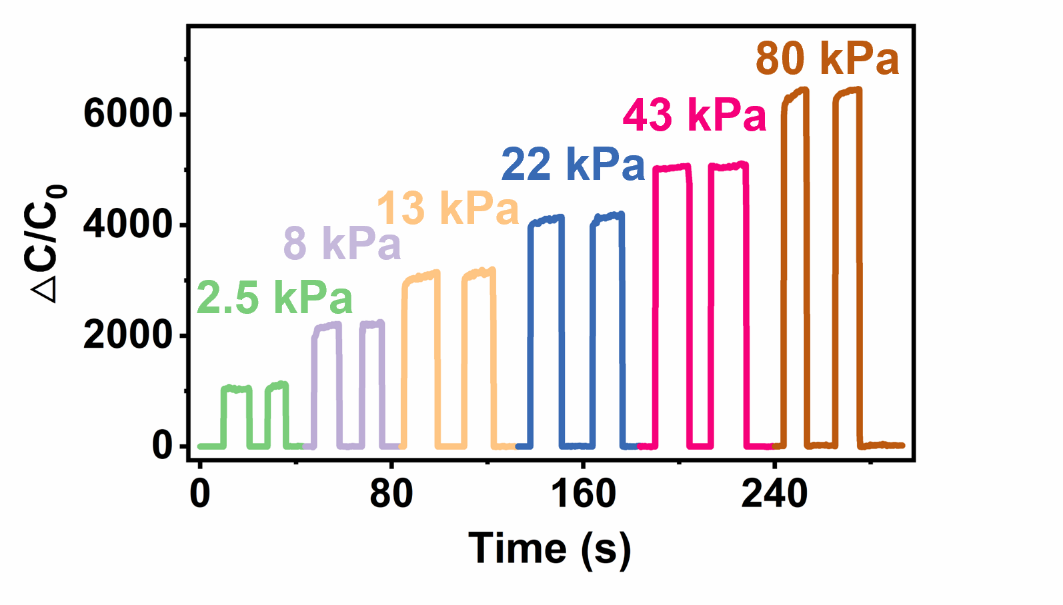


**Fig. S5** Response of device under repeated different pressures (2.5 kPa, 8 kPa, 13 kPa, 22 kPa, 43 kPa, and 80 kPa)


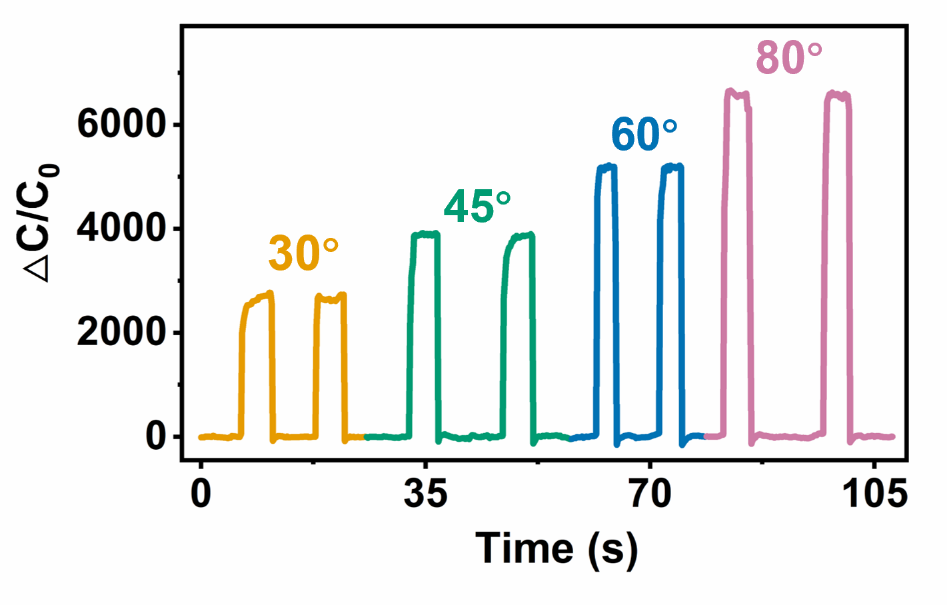


**Fig. S6** Response of device at different bending angles (30°, 45°, 60°, and 80°)


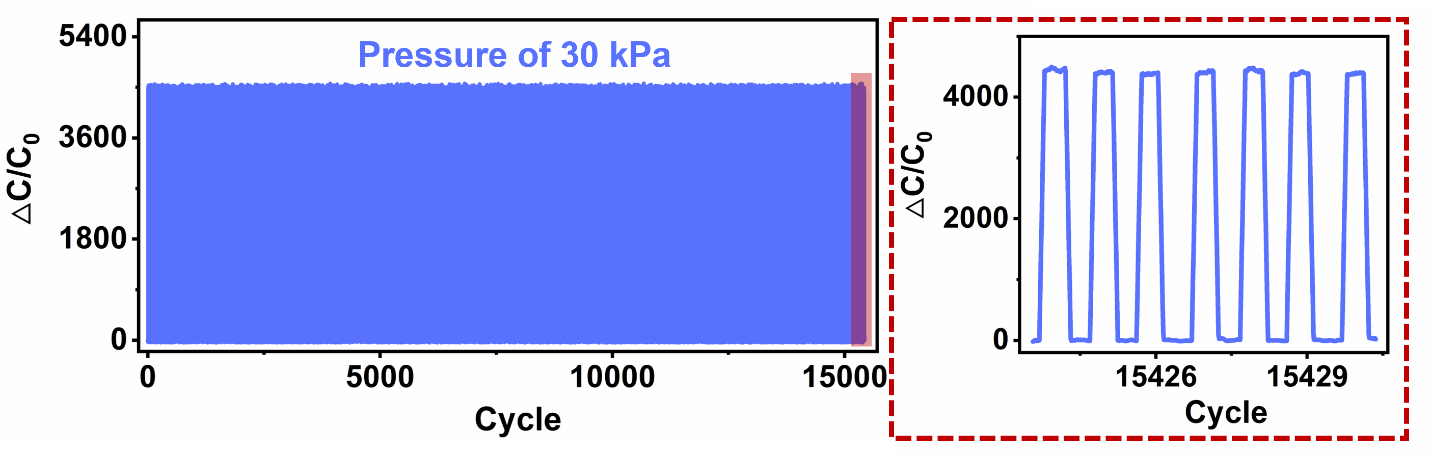


**Fig. S****7** Durability test of device over 15,000 cycles under pressure of 30 kPa, and inset showing the waveforms of the last few cycles

**
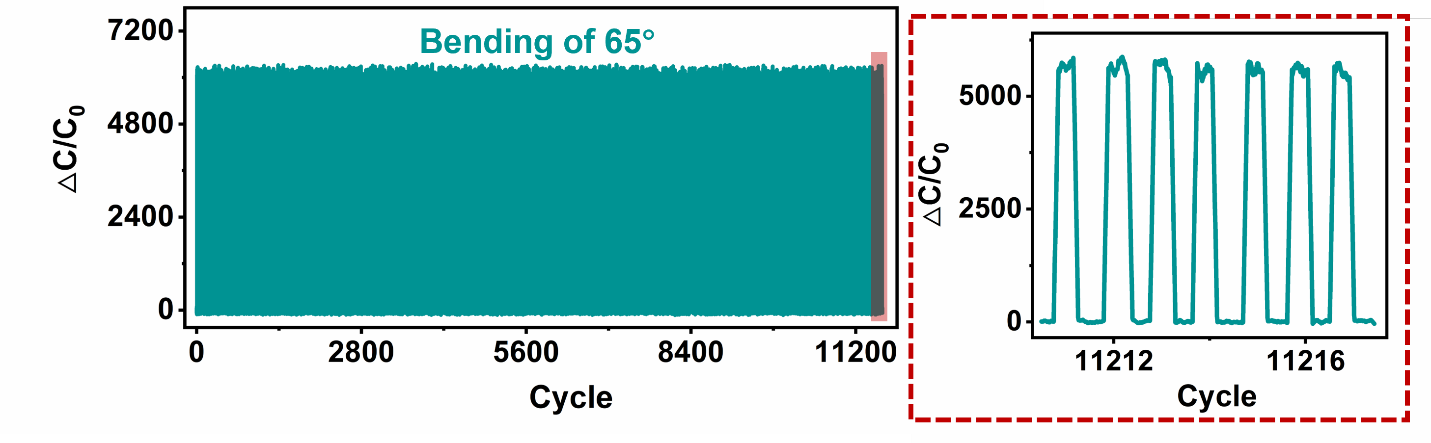
**

**Fig. S8** Durability test of device over 11,000 cycles of bending at 65°, and inset shows the last few cycle waveforms

**
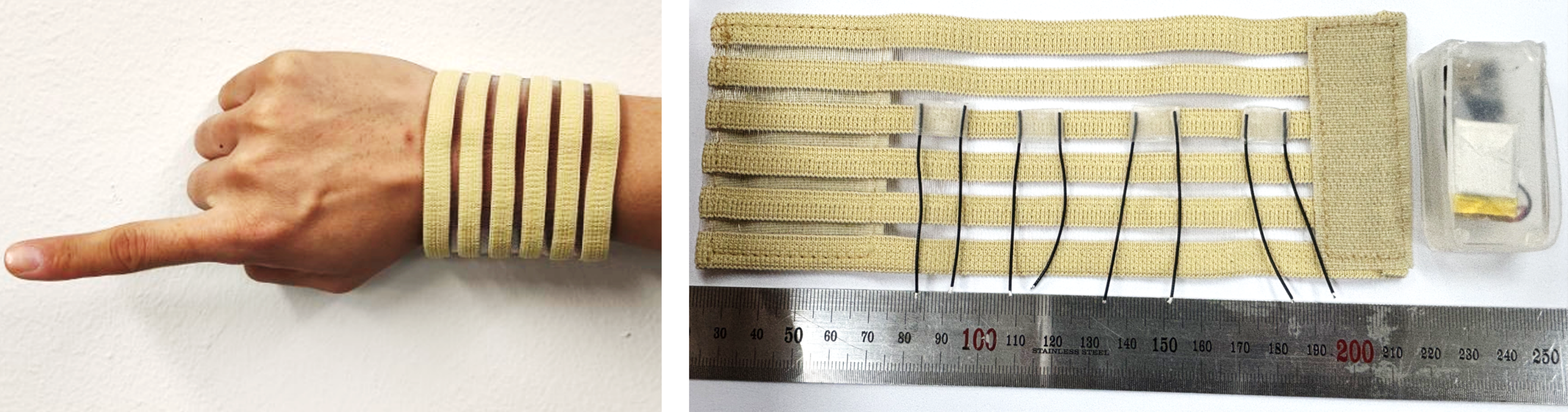
**

**Fig. S9** Photo of the wearable wristband system


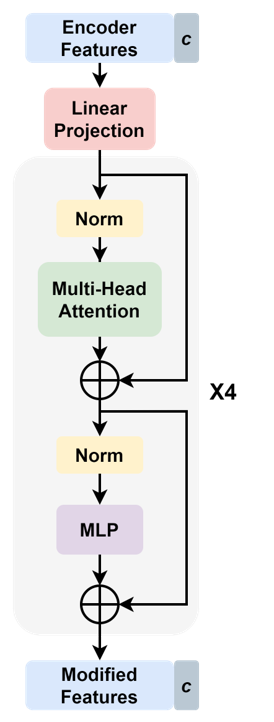


**Fig. S10** Detailed architecture of Transformer in cross-view fusion module (Token c is added to input features of model as representative vector)

**
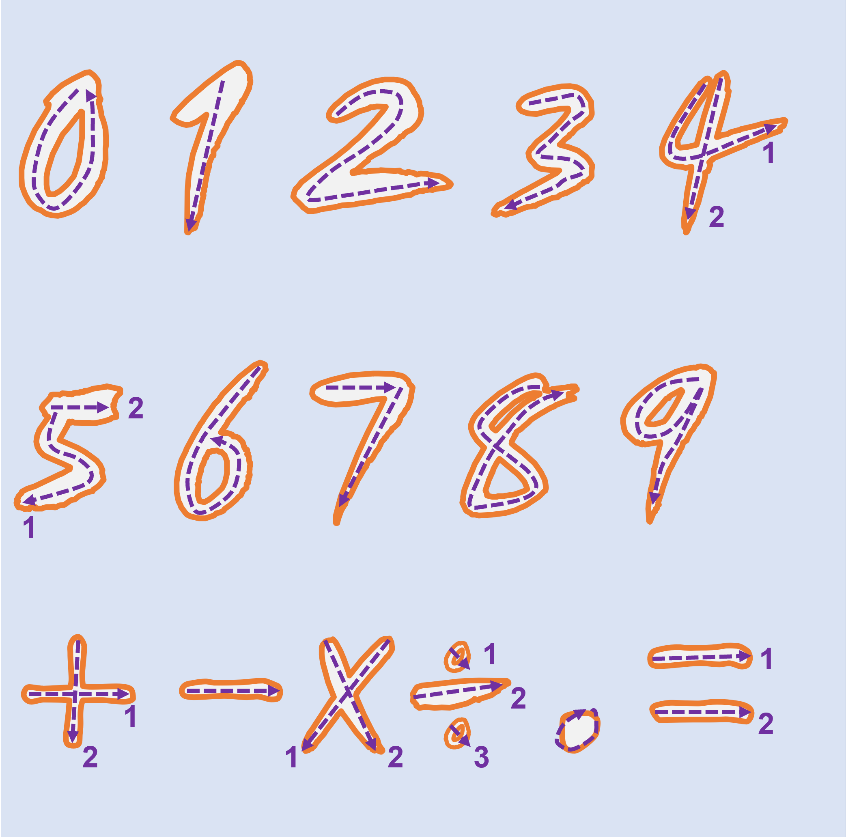
**

**Fig. S11** Sequence of air-writing trajectory of numbers “0–9” and six symbols

**
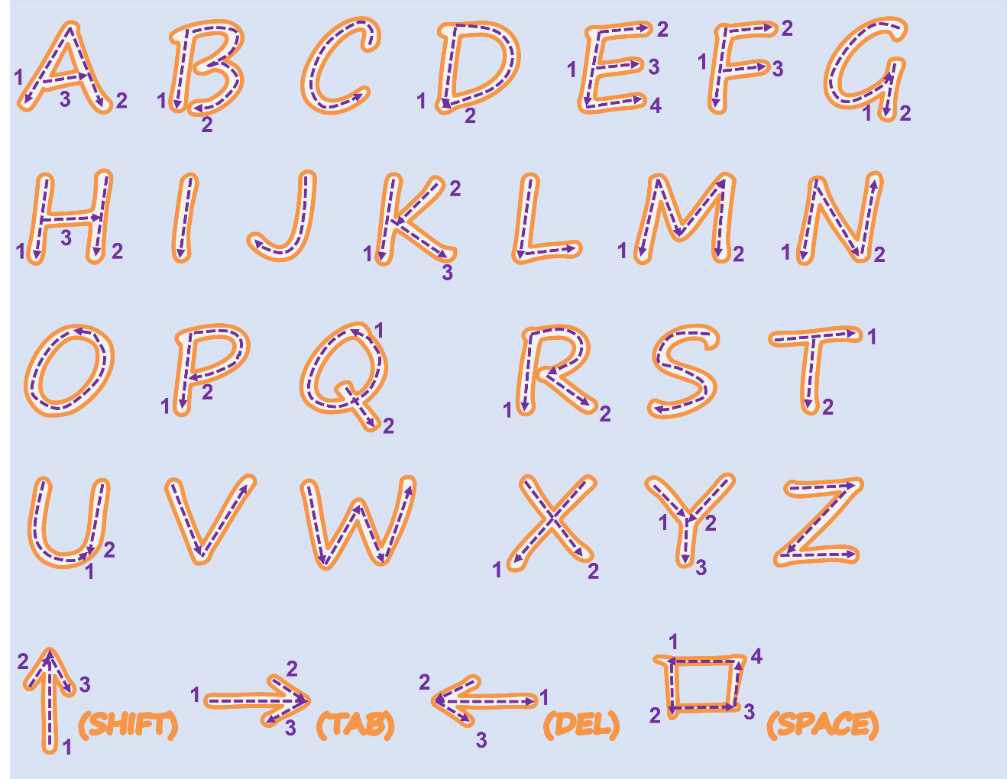
**

**Fig. S1****2** Sequence of air-writing trajectory of letters “A–Z” and four functional keys


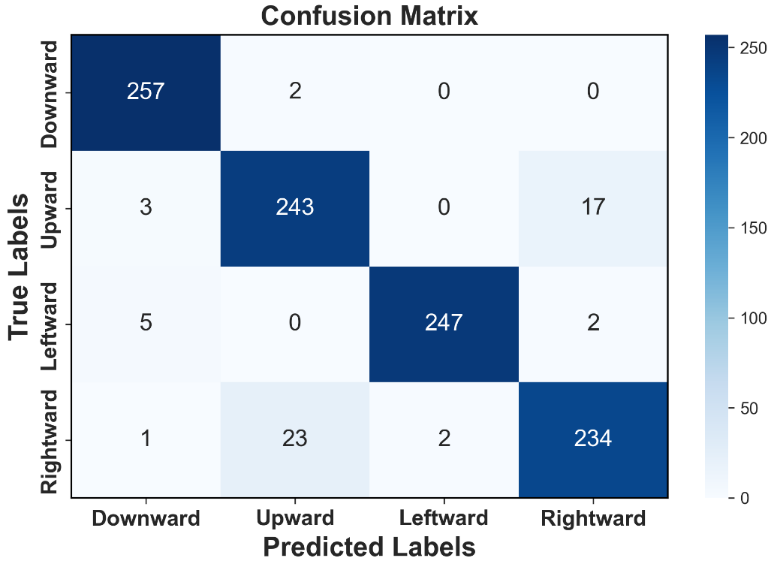


**Fig. S13** Confusion matrix for prediction of four directions, with an average accuracy of 94.7%


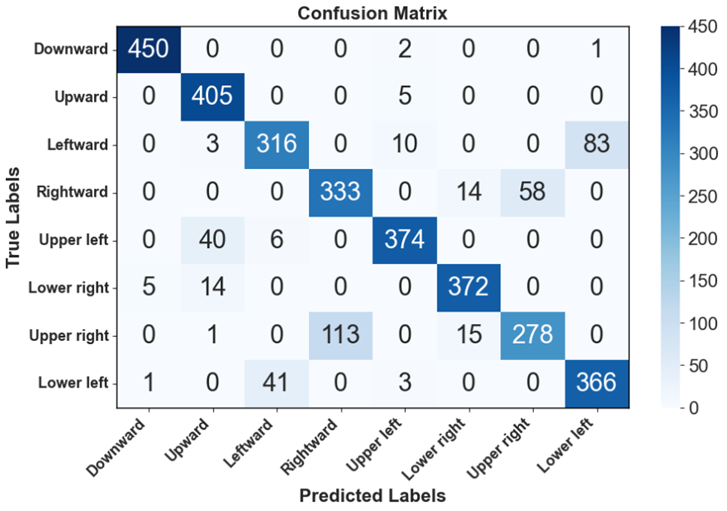


**Fig. S14** Confusion matrix for prediction of eight directions with 10-shot, showing an average accuracy of 87.5%

**
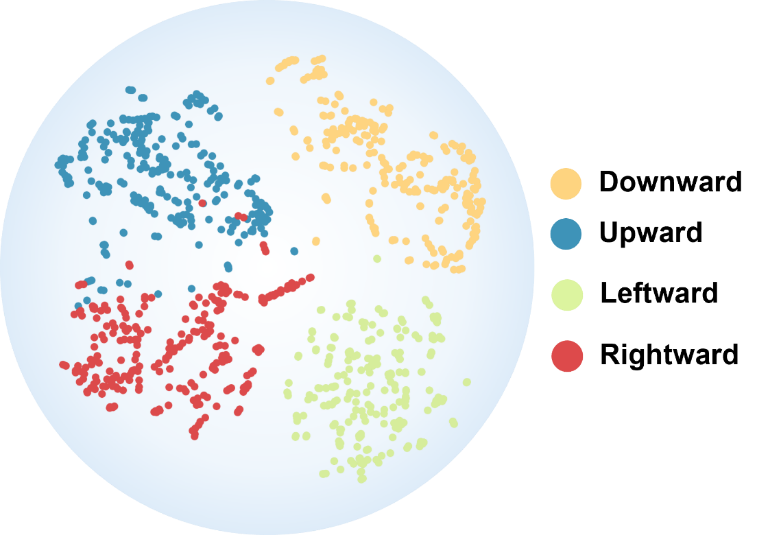
**

**Fig. S15** t-SNE projection of high-dimensional latent features of labeled data for four-direction-recognition using transfer learning

**
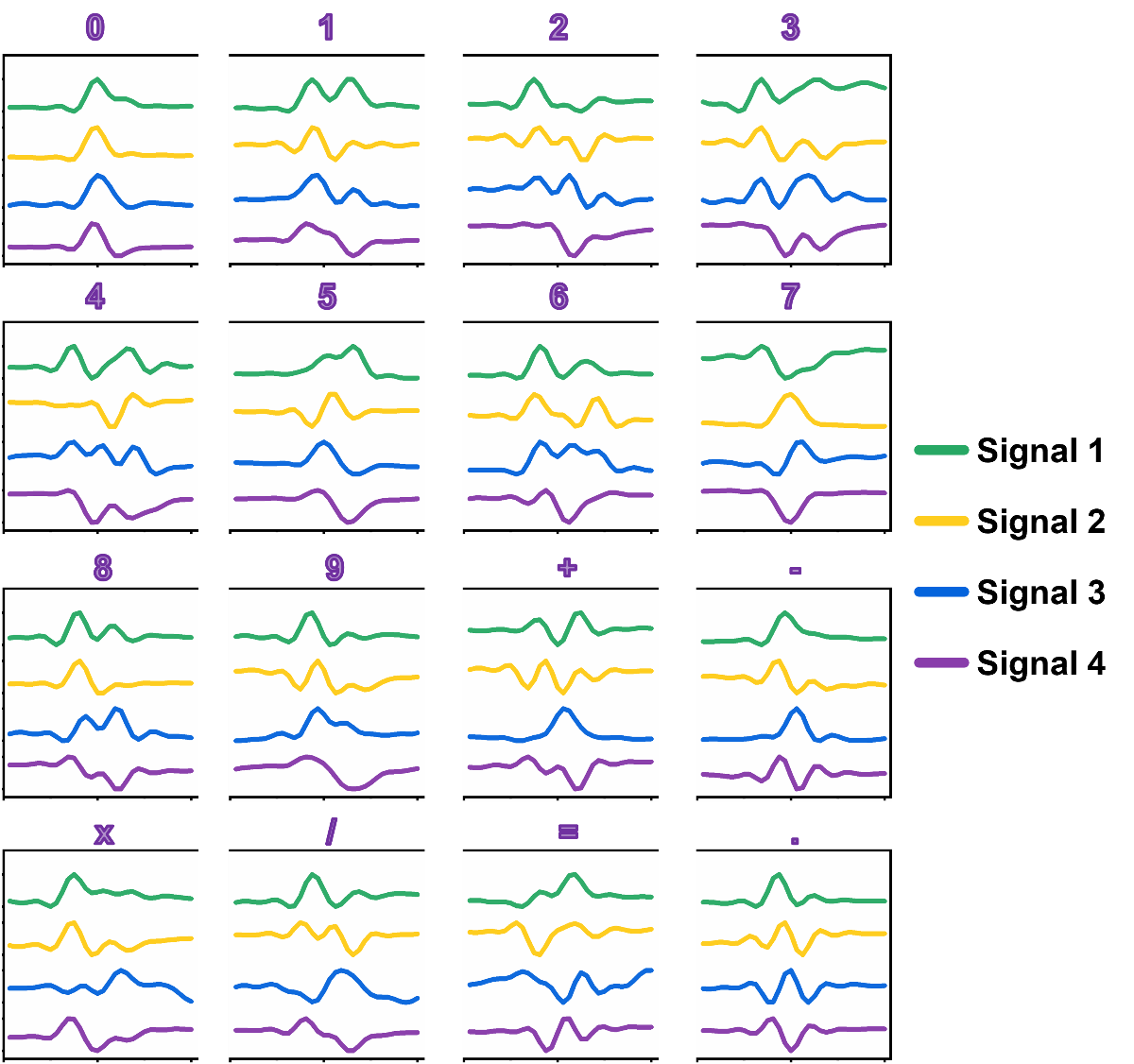
**

**Fig. S1****6** Waveforms of real-time four-channel signals of numbers “0–9” and six symbols

**
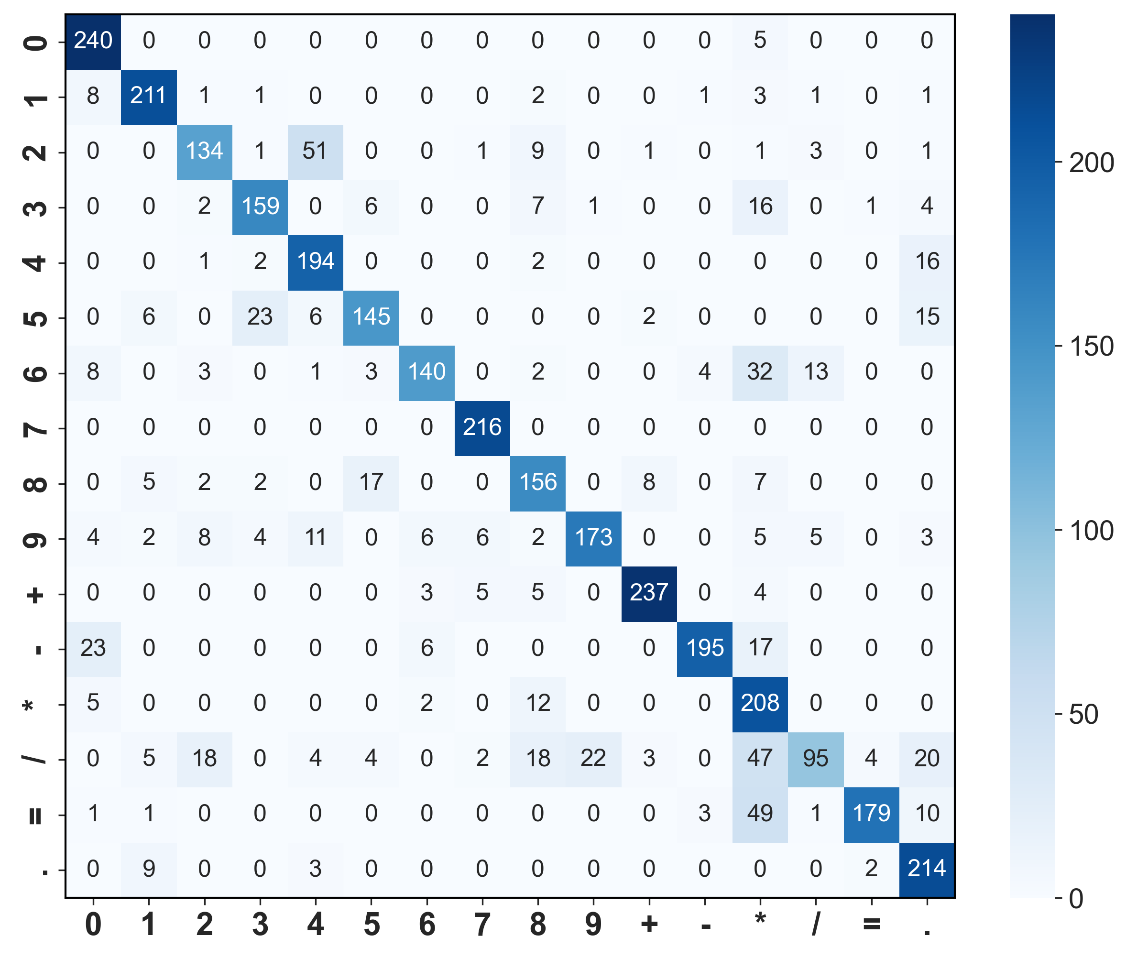
**

**Fig. S17** Confusion matrix for prediction of numbers, with an average accuracy of 81.2%

**
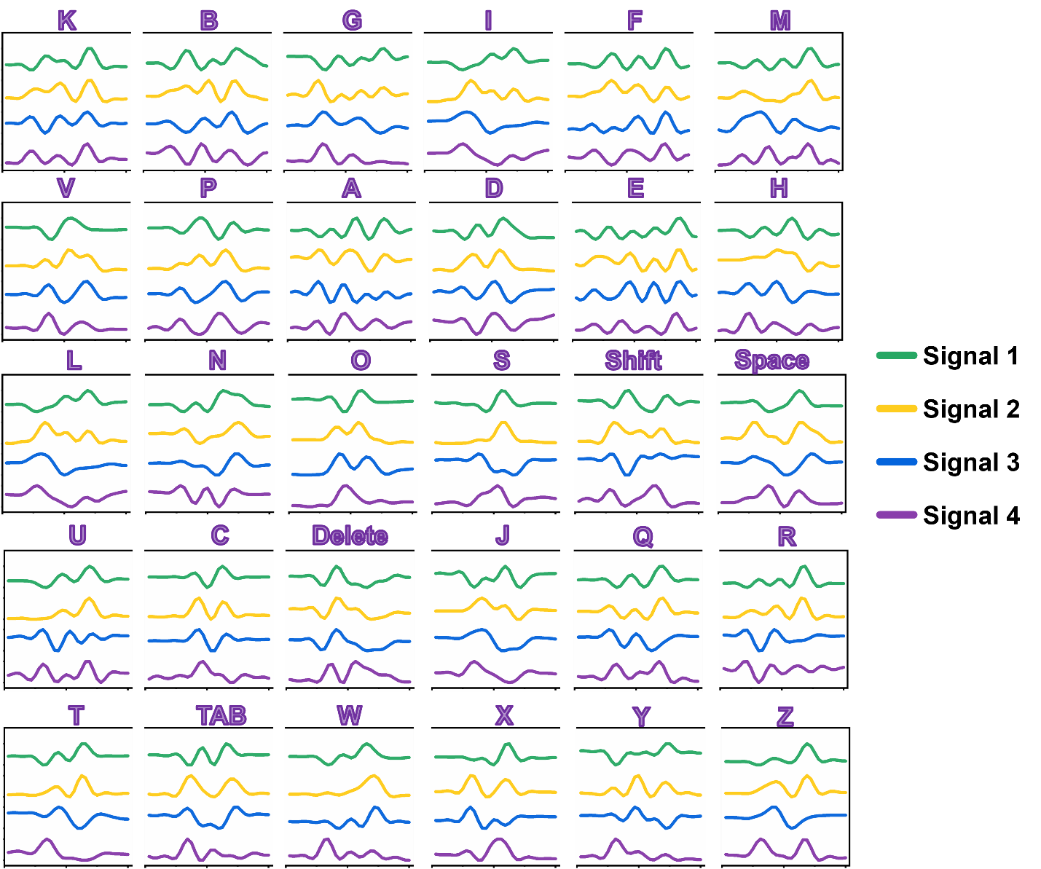
**

**Fig. S18** Waveforms of real-time 4-channel signals of letters “A–Z” and four functional keys

**
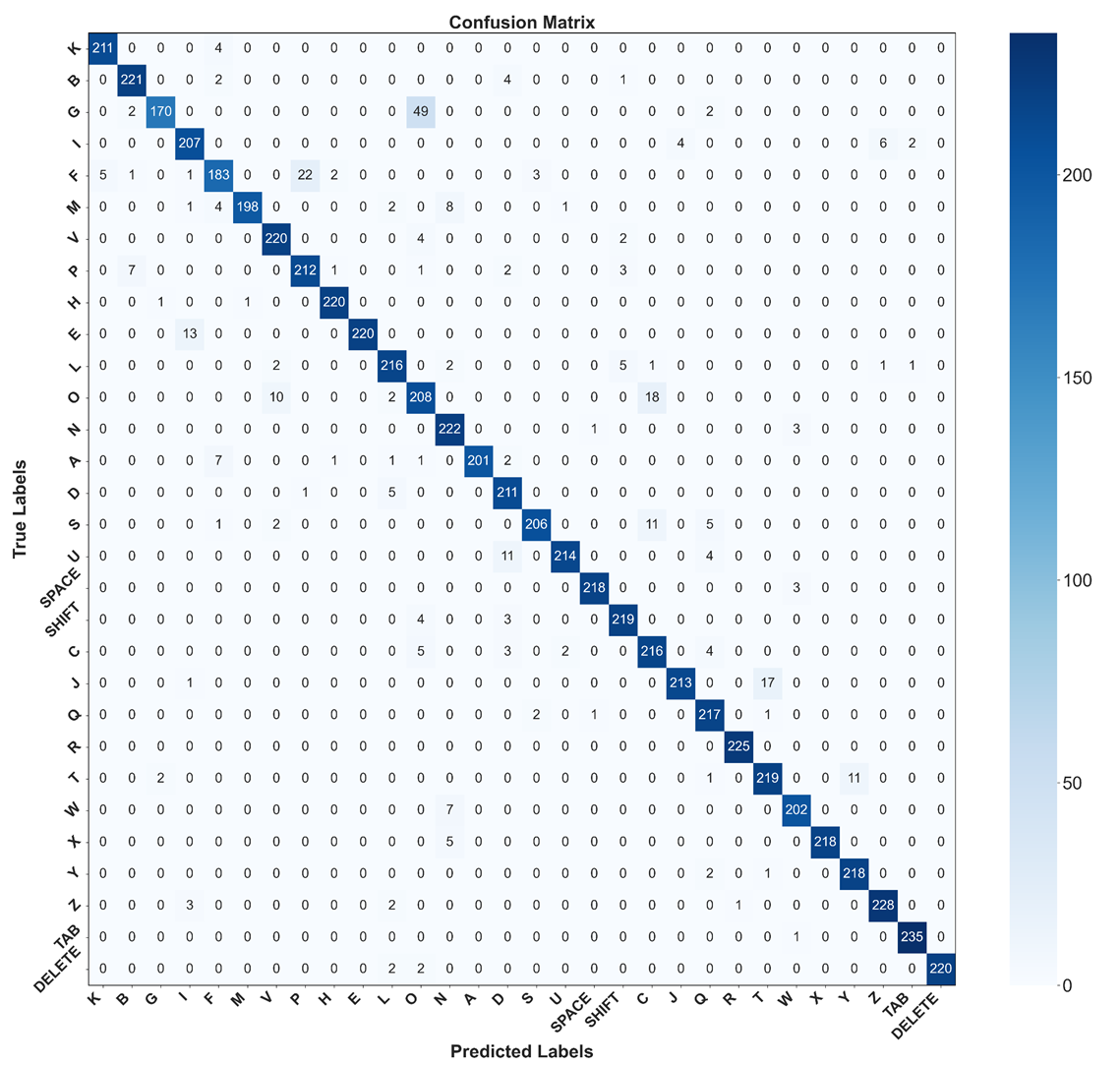
**

**Fig. S1****9** Confusion matrix for user 1’s air-writing 26 letters and four function keys, with an average accuracy of 94.9%

**
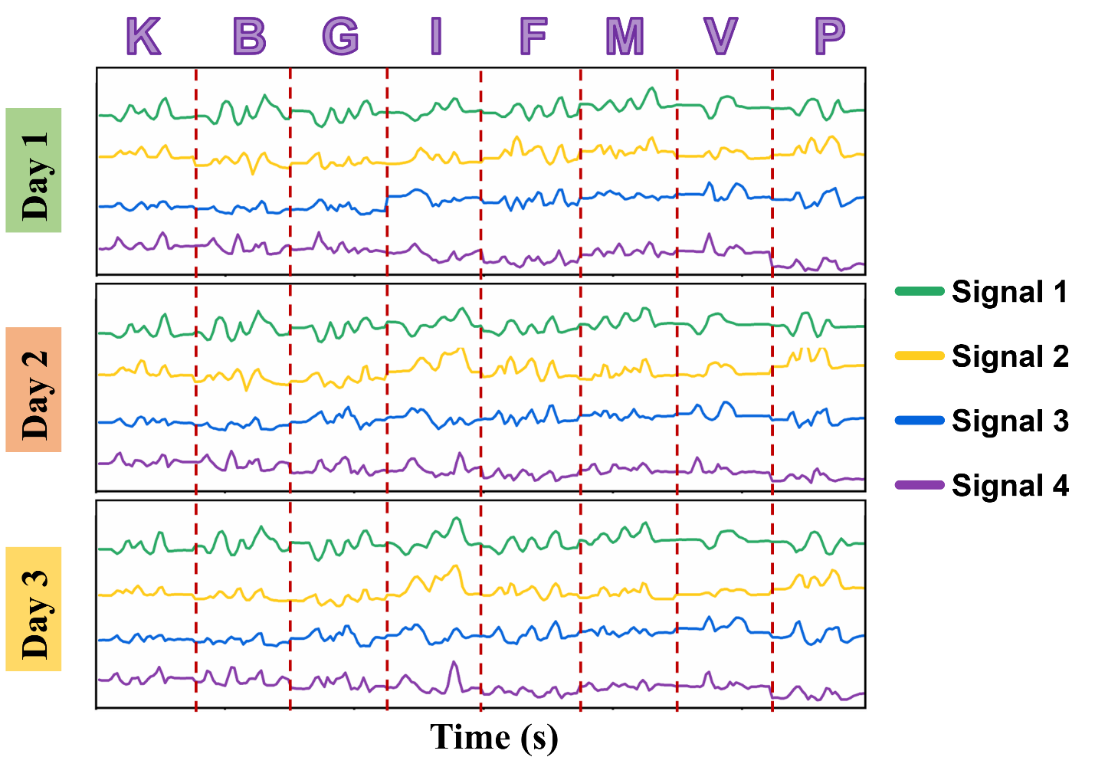
**

**Fig. S****20** Four-channel signal changes corresponding to letters “K B G I F M V P,” air-written by the same user at different times


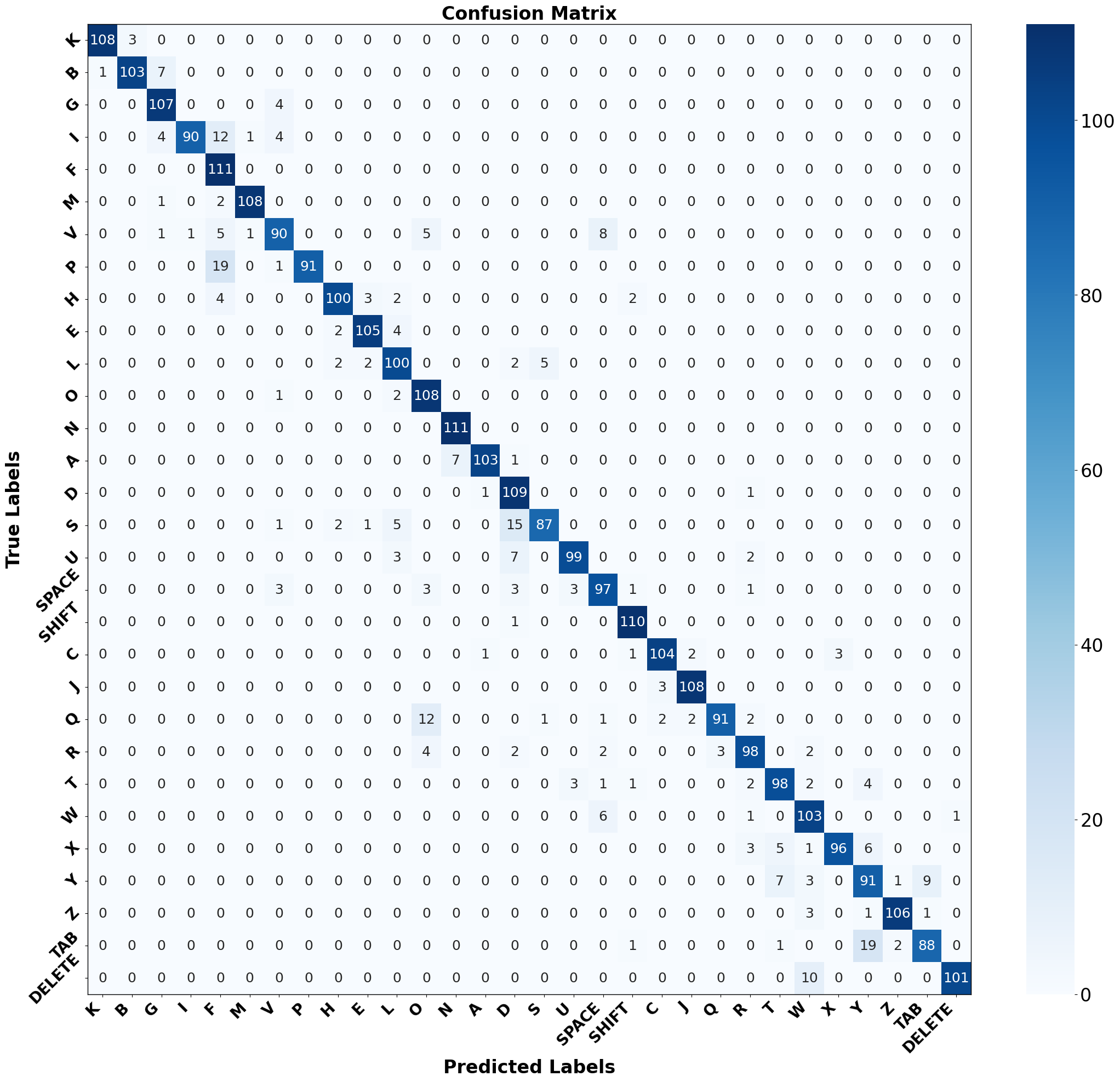


**Fig. S21** Confusion matrix for user 1’s air-writing 26 letters and four function keys on the second day, showing an average accuracy of 90.7%


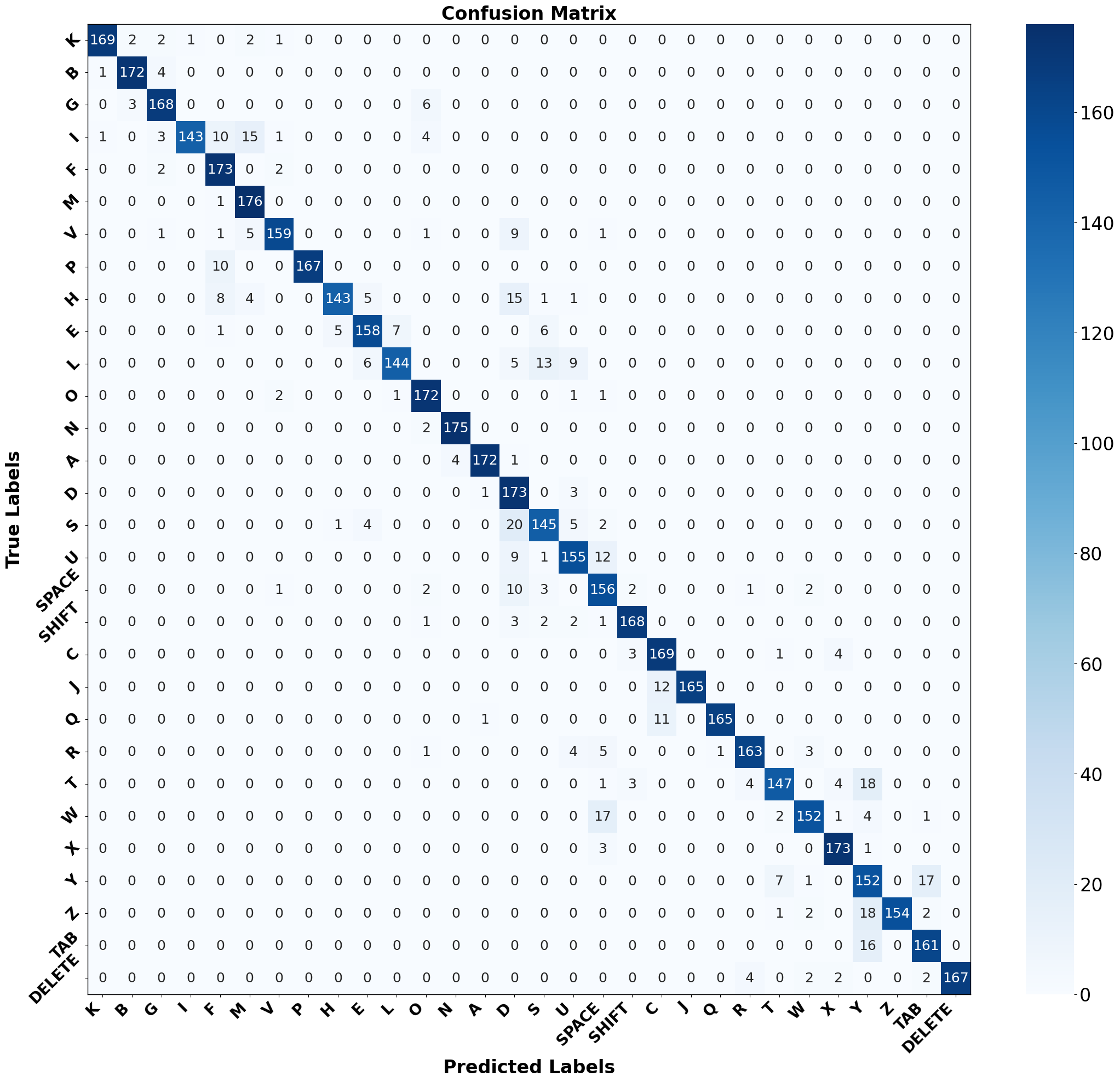


**Fig. S22** Confusion matrix for user 1’s air-writing 26 letters and four function keys on the third day, showing an average accuracy of 91.5%


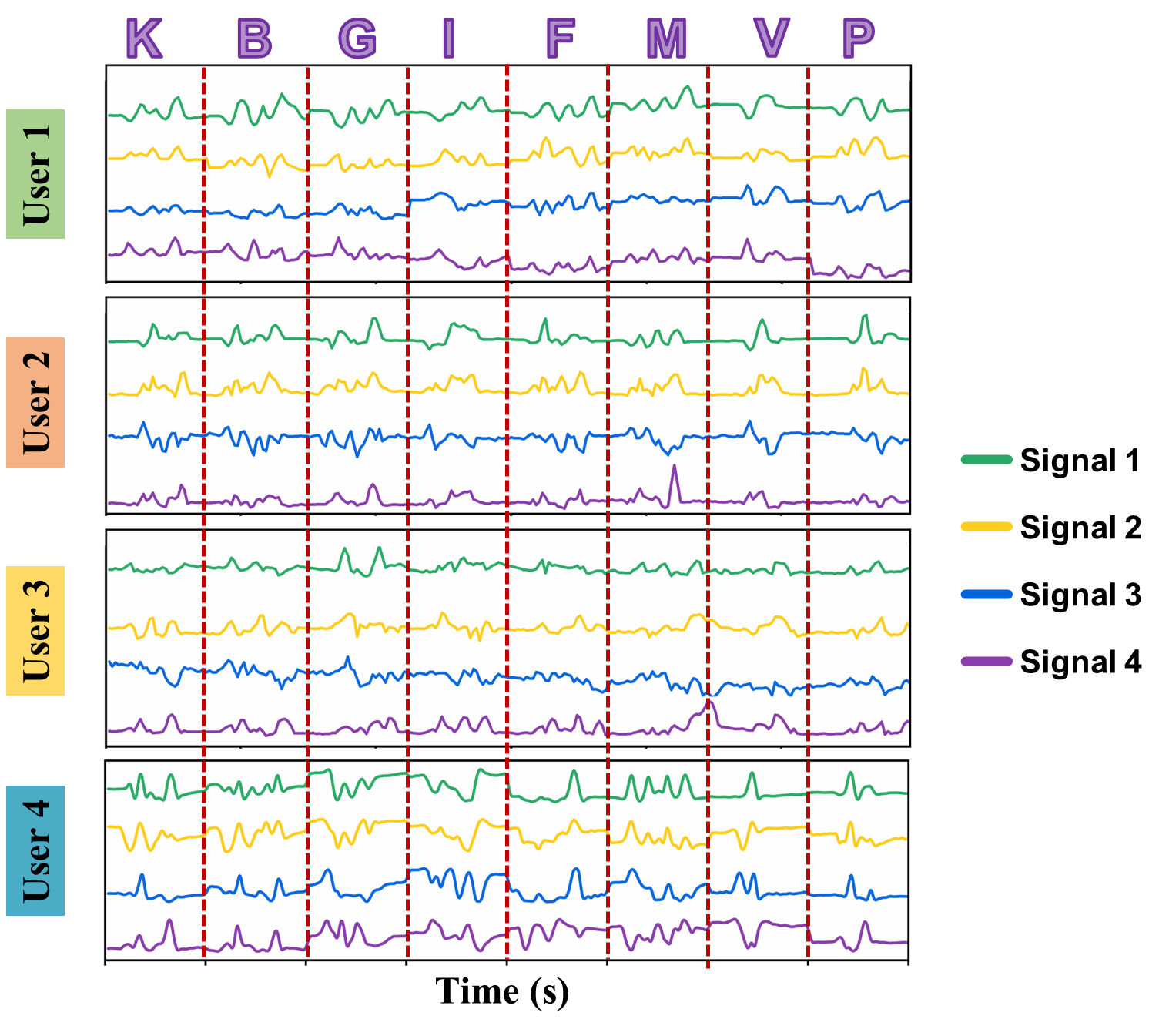


**Fig. S23** Four-channel signal changes corresponding to letters “K B G I F M V P,” air-written by four different users


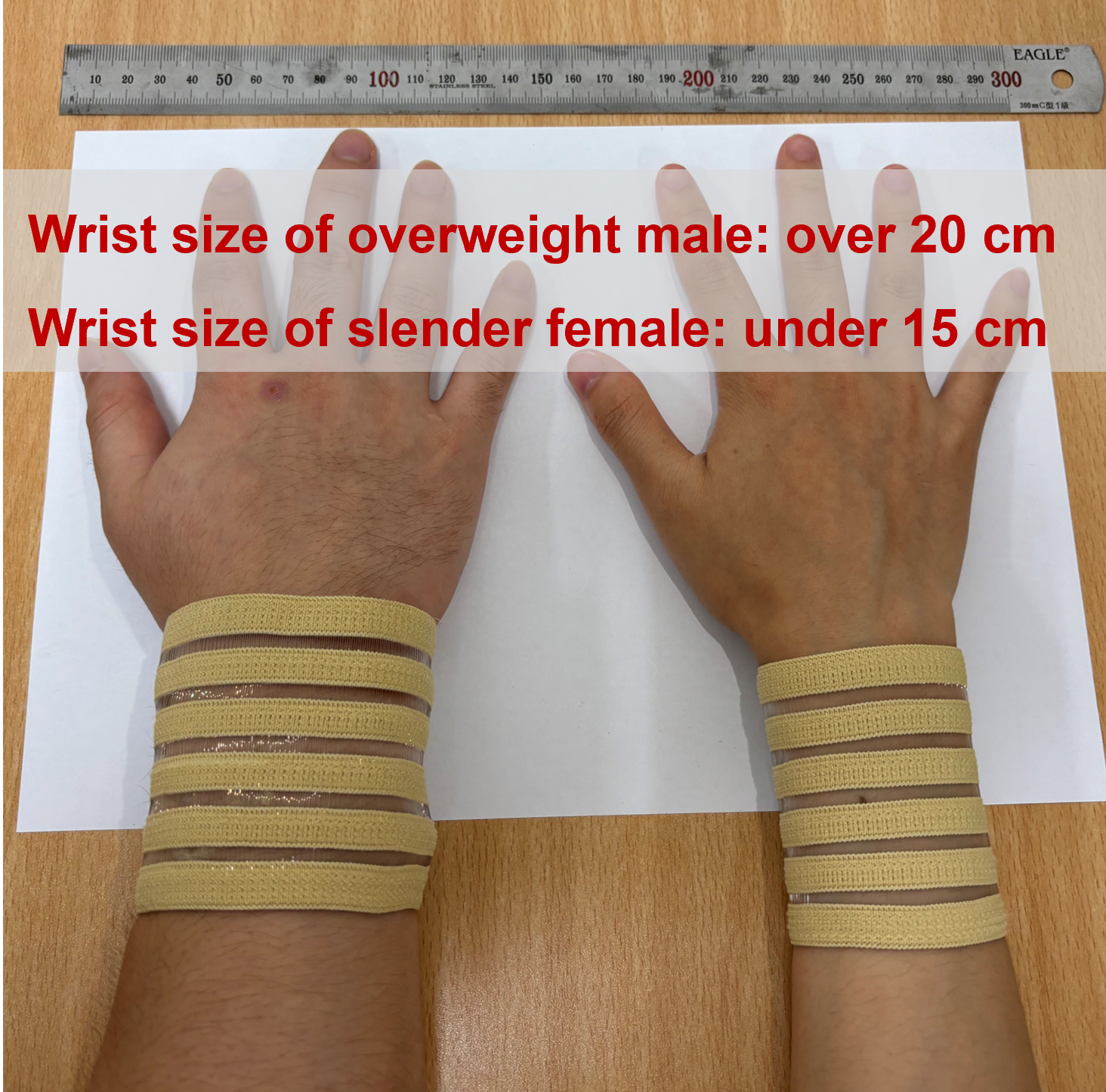


**Fig. S24** Photos of relatively thinner wrist and thicker wrist wearing the wristbands


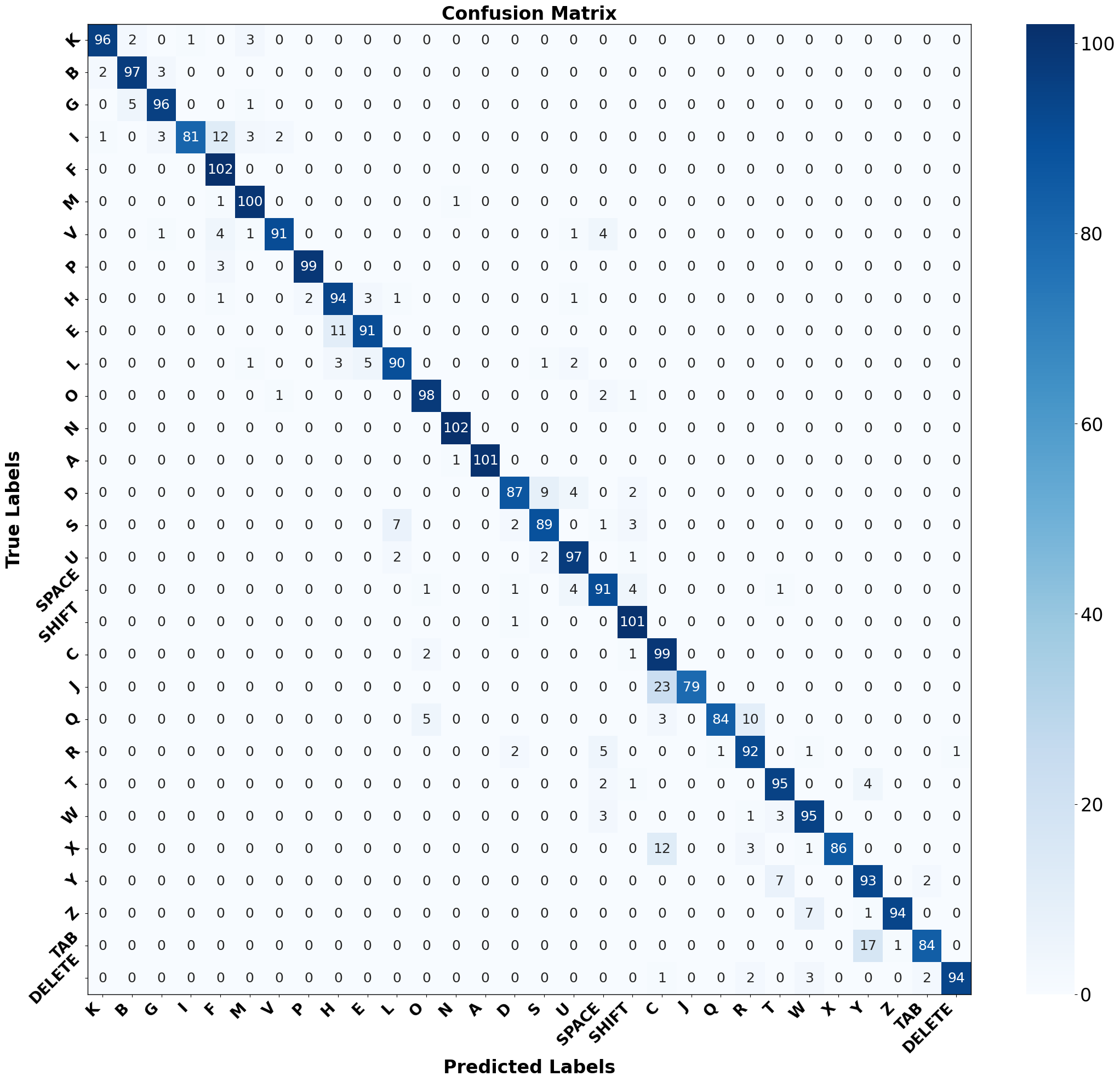


**Fig. S25** Confusion matrix for user 2’s air-writing 26 letters and four function keys, with an average accuracy of 91.4%


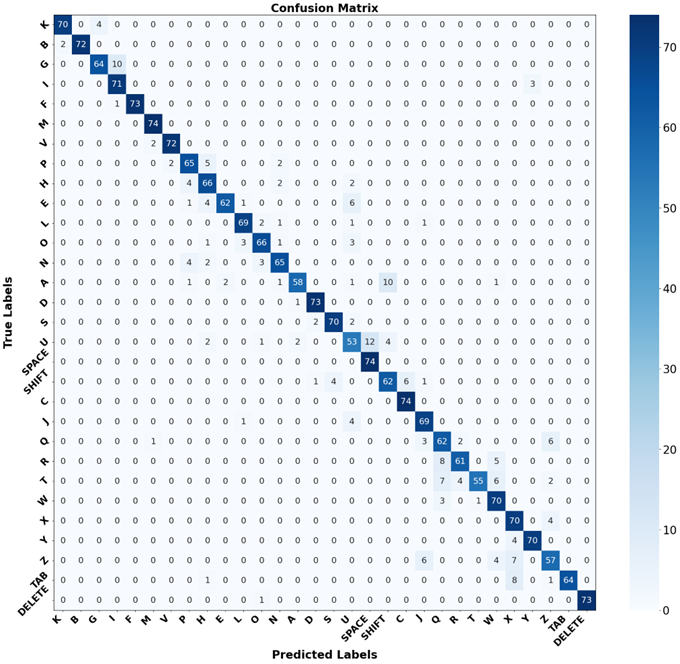


**Fig. S26** Confusion matrix for user 3’s air-writing 26 letters and four function keys, with an average accuracy of 90.3%


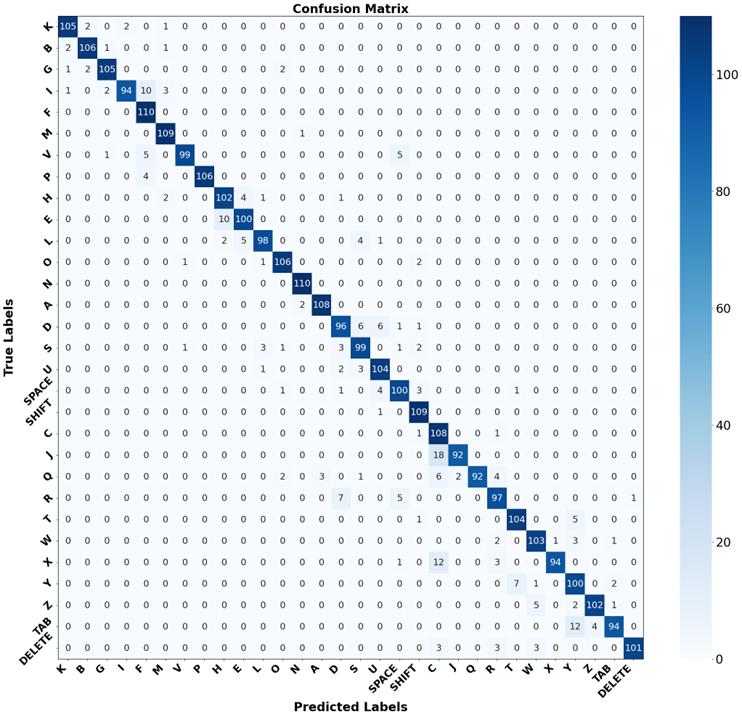


**Fig. S27** Confusion matrix for user 4’s air-writing 26 letters and four function keys, with an average accuracy of 92.5%


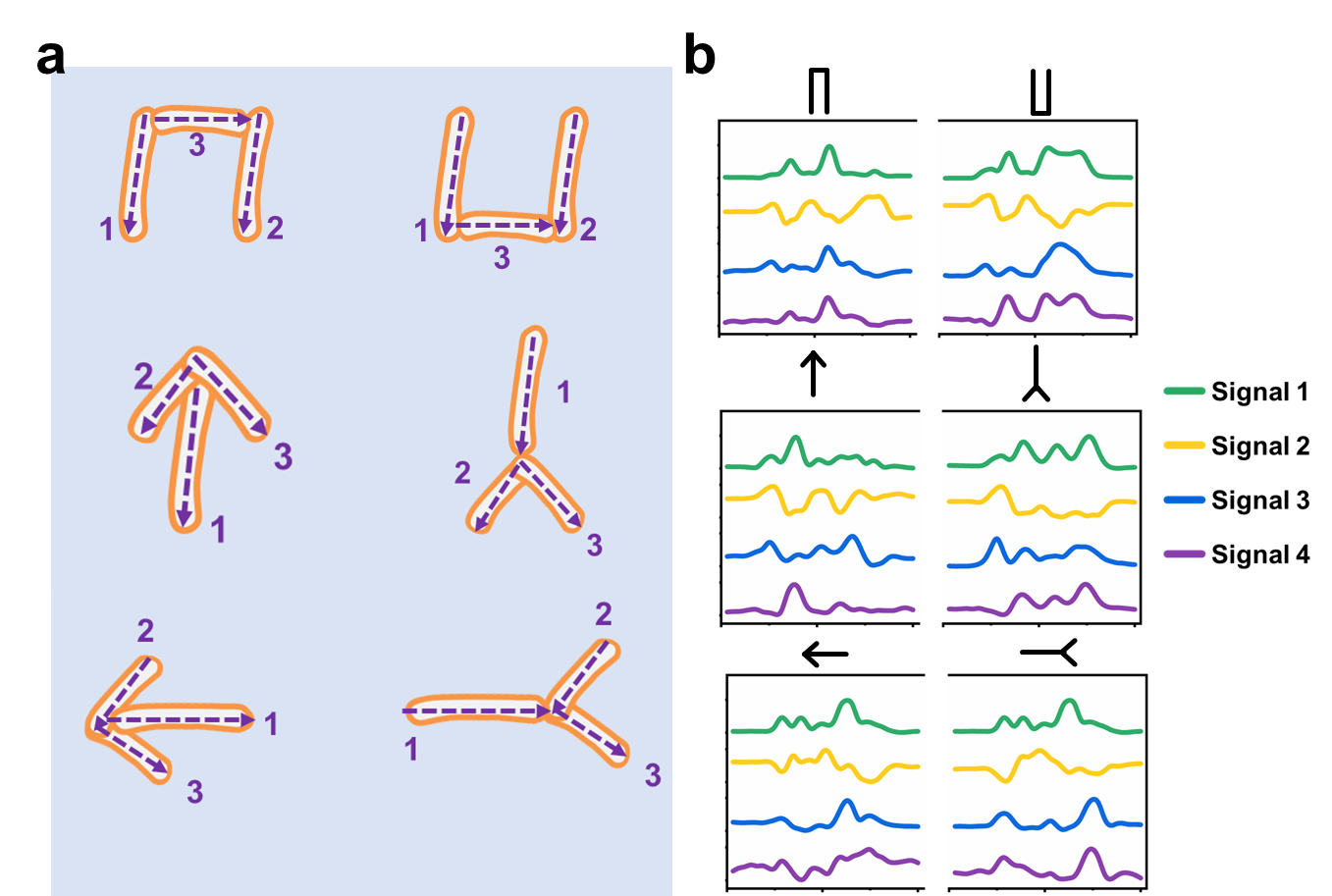


**Fig. S28 a** Sequence of air-writing trajectory of symbols with identical strokes arranged in different relative positions, and **b** corresponding four-channel signals


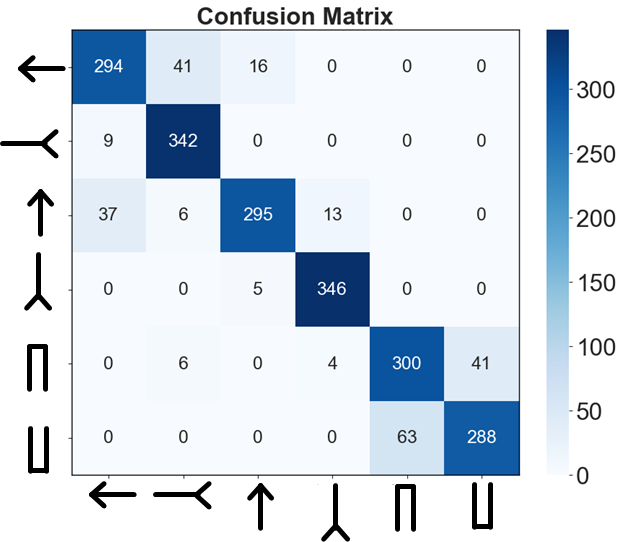


**Fig. S29** Confusion matrix for symbols with identical strokes arranged in different relative positions, showing an accuracy of 88.6%


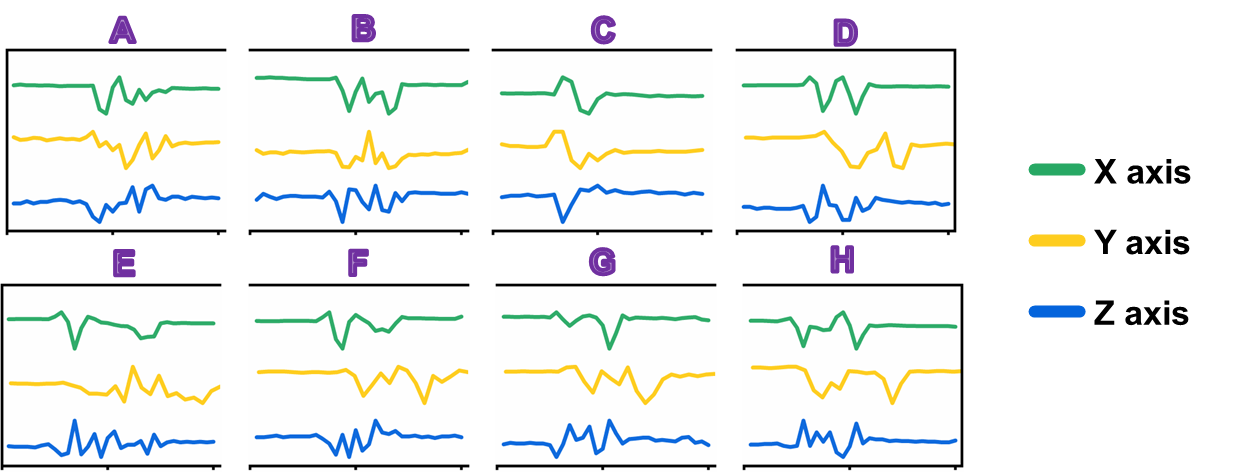


**Fig. S30** Signal plots of the X, Y, and Z axes when air-writing the letters A to H using the accelerometer


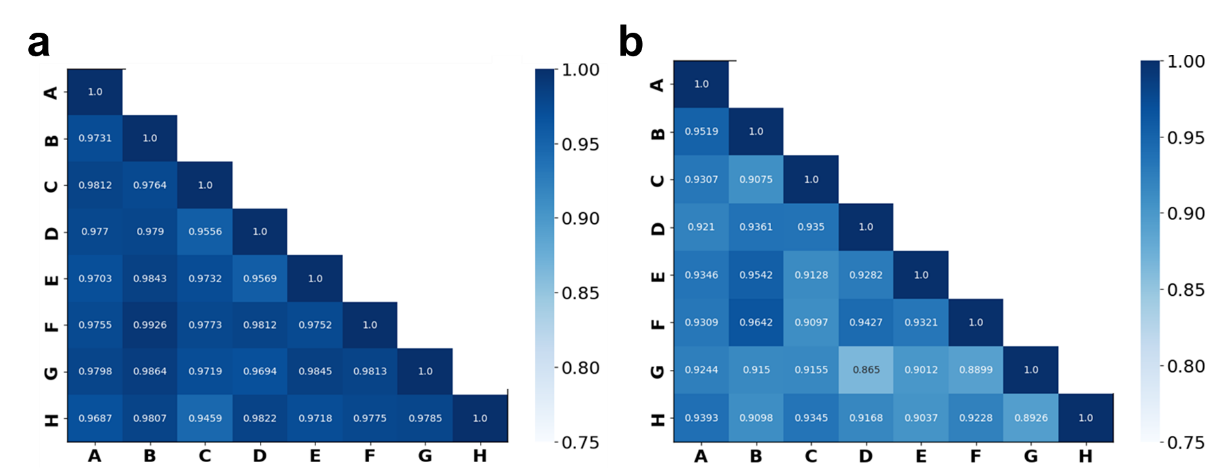


**Fig. S31** Similarity matrices for signals from **a**) the accelerometer and **b**) the flexible sensing devices when air-writing the letters A to H


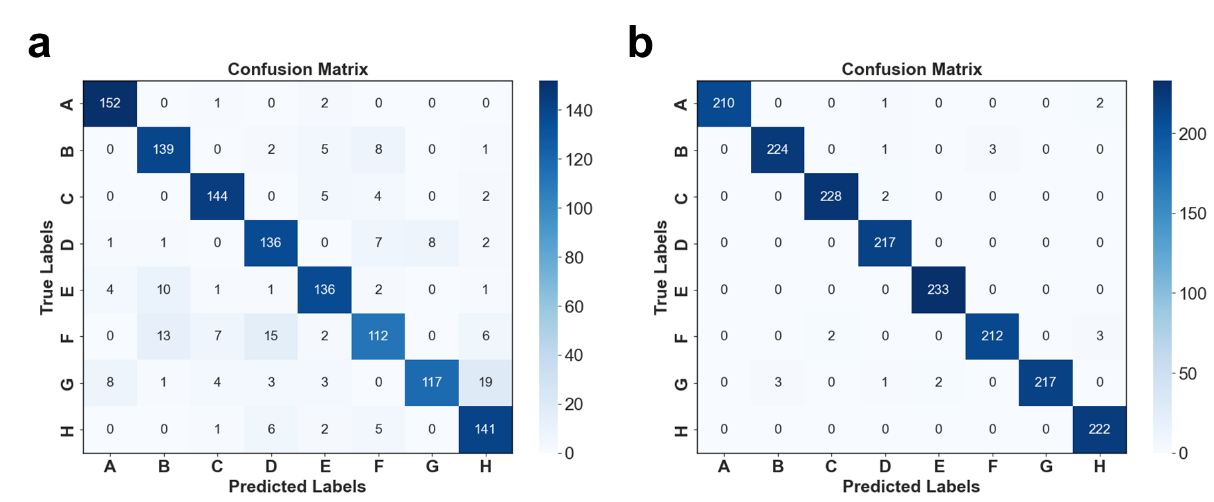


**Fig. S32** Confusion matrixes for air-writing letters A to H using **a**) the accelerometer and **b**) flexible capacitive sensors, with an average accuracy of 86.9% and 98.9%, respectively


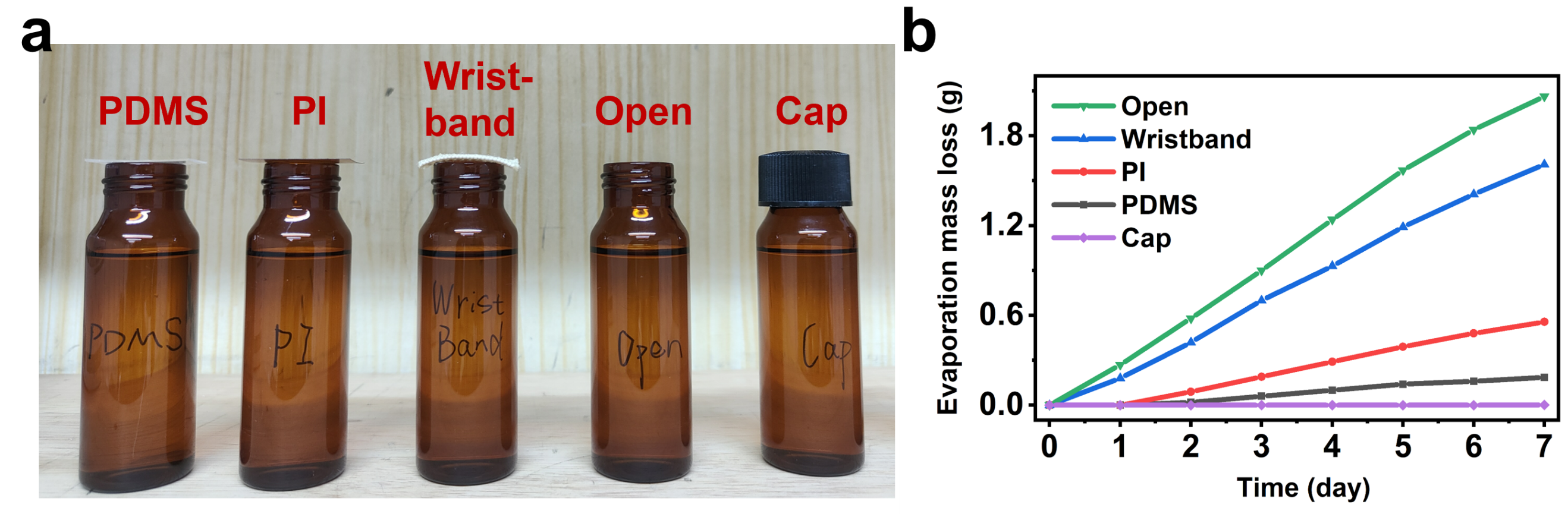


**Fig. S33 a** Optical image of the water vapor permeability test for the water-containing bottles covered with different materials, including Polydimethylsiloxane (PDMS) film, polyimide (PI) film, wristband material, a sealed bottle with a cap, and an open bottle. **b** Mass loss curves of the water contained in the bottles

**S3 Supplementary Tables**

**Table S1** Power consumption of Wi-Fi module

| **Parameters** | **Typical** | **Unit** |
| --- | --- | --- |
| Tx 802.11b, CCK 11Mbps, P OUT=+17dBm | 170 | mA |
| Tx 802.11g, OFDM 54Mbps, P OUT=+15dBm | 140 | mA |
| Tx 802.11n, MCS7, P OUT=+13dBm | 120 | mA |
| Rx 802.11b, 1024 bytes packet length, -80dBm | 50 | mA |
| Rx 802.11g, 1024 bytes packet length, -70dBm | 56 | mA |
| Rx 802.11n, 1024 bytes packet length, -65dBm | 56 | mA |
| Modem-Sleep | 15 | mA |
| Light-Sleep | 0.9 | mA |
| Deep-Sleep | 10 | uA |

**Table S2** Ablation experiment study on the effects of different data augmentation methods in number and letter prediction

| **Strong augmentation** | **Weak augmentation** | **Number** **accuracy** | **Letter accuracy** |
| --- | --- | --- | --- |
| no aug | scale | 74.4 | 79.5 |
| no aug | scale + jitter | 75.9 | 83.0 |
| permutation | no aug | 75.3 | 82.6 |
| jitter + permutation | no aug | 77.0 | 86.6 |
| jitter + permutation | time shift | 78.6 | 86.4 |
| jitter + permutation | time shift + jitter | 76.6 | 85.1 |
| jitter + permutation | scale | 79.1 | 90.1 |
| **jitter + permutation** | **scale + jitter** | **81.2** | **94.9** |

**Table S3** Ablation experiments of effect of different components in TS-VFC model

| **Component** | **Prediction of 8 directions** | **Prediction of numbers** | **Prediction of**  **letters** |
| --- | --- | --- | --- |
| TS-C^a)^ | 79.8 | 76.9 | 92.7 |
| TS-VC^b)^ | 80.6 | 76.0 | 92.6 |
| TS-VFC (*ours*) | **82.0** | **81.2** | **94.9** |
| TS-VFC (Weak only) | 80.9 | 74.8 | 91.4 |
| TS-VFC (Strong only) | 80.1 | 72.4 | 90.7 |

^a)^TS-C represents that Transformer structure is not used, and projection head is directly connected.

^b)^TS-VC represents the use of the Transformer structure and does not use cross-view fusion module.

Moreover, we show the impact of using two weak or strong augmentation on its performance. It is evident that a combination of weak and strong augmentation yields the best performance.

**Table S4** Detailed structure of the encoder

| **Layer name** | **Operator** | **Kernel size** | **Padding** | **Stride** | **Input**  **Channel** | **Input**  **Size** | **Channel**  **size** | **Output**  **Size** |
| --- | --- | --- | --- | --- | --- | --- | --- | --- |
| **Conv 1** | 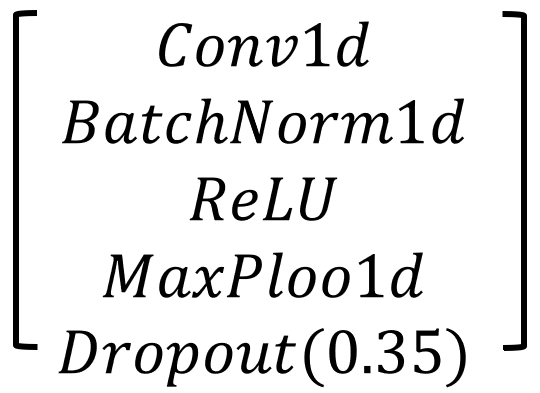 | 4 | 2 | 1 | 4 | 4×32 | 32 | 32×17 |
| **Conv 2** | 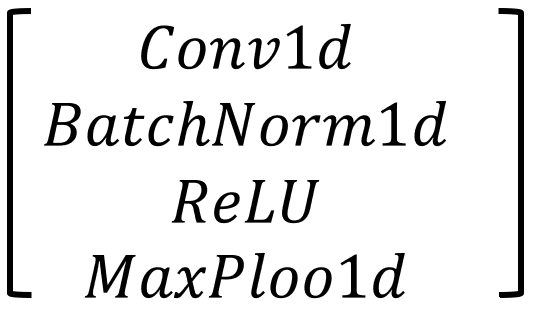 | 4 | 4 | 1 | 32 | 32×17 | 64 | 64×12 |
| **Conv 3** | 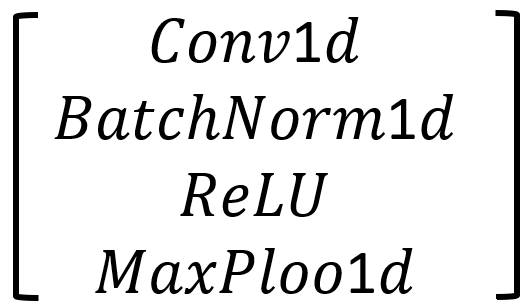 | 4 | 4 | 1 | 64 | 64×12 | 128 | 128×9 |

The encoder is composed of three layers of one-dimensional convolutional neural network, which extract two-dimensional features from time-seires signals.

**Table S5** Test results after fine-tuning the TS-VFC model with 3 or 5 labels

| **Task** | **Accuracy** | **Precision** | **Recall** | **MF1-score** |
| --- | --- | --- | --- | --- |
| Prediction of four directions | 94.7 | 94.7 | 94.7 | 94.7 |
| Prediction of eight directions | 82.0 | 83.1 | 81.8 | 81.4 |
| Prediction of numbers | 81.2 | 83.0 | 81.0 | 80.7 |
| Prediction of letters | 94.9 | 95.1 | 94.9 | 94.9 |

**Table S6** Representative study on task performance using deep learning-enabled wearable devices

| **Sensor type** | **Learning**  **objectives** | **Dynamic gestures** | **Learning**  **method** | **Dataset** | **Multi-task**  **support** | **Multi-user**  **support** | **Number of sensors** | **Accuracy** |
| --- | --- | --- | --- | --- | --- | --- | --- | --- |
| Piezoresistive sensor [S1] | 12 hand gestures | **×** | Supervised learning (SVM) | Labeled 120 shots | **×** | **√** | 5 | 96.3% |
| Triboelectric and piezoelectric sensor [S2] | 26 hand gestures | **×** | Supervised learning (LDA) | **○** | **×** | **○** | 8 | 92.6% |
| Triboelectric sensor [S3] | 21hand gestures | **×** | Supervised learning (CNN) | Labeled 7350  shots | **×** | **○** | 7 | 97.6% |
| Optical-nanofiber-based sensor [S4] | 21hand gestures | **×** | Supervised learning (SVM) | **○** | **×** | **√** | 3 | 94.0% |
| Surface electromyography and inertial measurement unit [S5] | 8 air gestures and 4 surface | **√** | Supervised learning (LDA) | **○** | **×** | **√** | 5 | 92.6% |
| Capacitive sensor [S6] | 3 gestures | **×** | Supervised learning (SVM) | **○** | **×** | **√** | 5 | 90.0% |
| Barometric sensor [S7] | 6 wrist gestures, 5 finger gestures, and 10 Chinese number gestures | **×** | Supervised learning (LDA or SVM) | Labeled 2500  shots per task | **√** | **√** | 10 | 94.0% |
| Substrate-less nanomesh [S8] | 4 Command,  Keyboard typing,  6 objects | **√** | Unsupervised Meta learning (Transformer) | Unlabeled random motions (900s), Few-shot Transfer (5-shots) | **√** | **√** | 1 | Numpad keys:85%,  Keyboard: 93%,  Objects:82% |
| **Iontronic sensor (This work)** | **8 directions, 10 numbers and 6 operators, 30 letters, and function keys** | **√** | **Unsupervised learning (CNN+**  **Transformer)** | **Unlabeled random motions (2000 s),**  **5-shot transfer** | **√** | **√** | **4** | **Direction:82.0%,Number: 81.2%, Letter: 94.9%** |

**Supplementary Movies**

**Movie S1** Real-time display of four-channel signals of random wrist movement, using wristband integrated wireless Wi-Fi module

**Movie S2** Learning and prediction of four directions

**Movie S3** Learning and prediction of full eight directions

**Movie S4** Direction control in a game using gesture commands

**Movie S5** Prediction of air-writing letters

**Movie S6** Prediction of air-writing numbers and calculation operations

**Movie S7** Virtual keyboard implemented by air-writing words

**Movie S8** Login system interface in three languages

**Supplementary References**

1. Y. Liu, X. Liang, H. Li, H. Deng, X. Zhang, et al., Ultralight smart patch with reduced sensing array based on reduced graphene oxide for hand gesture recognition. Adv. Intell. Syst. **4**, 2200193 (2022). <https://doi.org/10.1002/aisy.202200193>
2. P. Tan, X. Han, Y. Zou, X. Qu, J. Xue, et al., Self-Powered gesture recognition wristband enabled by machine learning for full keyboard and multicommand input. Adv. Mater. **34**, 2200793 (2022). <https://doi.org/10.1002/adma.202200793>
3. H. Fang, L. Wang, Z. Fu, L. Xu, W. Guo, et al., Anatomically designed triboelectric wristbands with adaptive accelerated learning for human–machine interfaces. Adv. Sci. **10**, 2205960 (2023). <https://doi.org/10.1002/advs.202205960>
4. S. Wang, X. Wang, S. Wang, W. Yu, L. Yu, et al., Optical‐Nanofiber‐Enabled gesture‐recognition wristband for human–machine interaction with the assistance of machine learning. Adv. Intell. Syst. **5**, 2200412 (2023). <https://doi.org/10.1002/aisy.202200412>
5. S. Jiang, B. Lv, W. Guo, C. Zhang, H. Wang, et al., Feasibility of wrist-worn, real-time hand, and surface gesture recognition via semg and imu sensing. IEEE Trans. Ind. Inf. **14**, 3376 (2018). <https://doi.org/10.1109/TII.2017.2779814>
6. X. Liang, R. Ghannam, H. Heidari, Wrist-Worn gesture sensing with wearable intelligence. IEEE Sensors J. **19**, 1082 (2019). <https://doi.org/10.1109/JSEN.2018.2880194>
7. P. B. Shull, S. Jiang, Y. Zhu, X. Zhu, Hand gesture recognition and finger angle estimation via wrist-worn modified barometric pressure sensing. IEEE Trans. Neural Syst. Rehabil. Eng. **27**, 724 (2019). <https://doi.org/10.1109/TNSRE.2019.2905658>
8. K. K. Kim, M. Kim, K. Pyun, J. Kim, J. Min, et al., A substrate-less nanomesh receptor with meta-learning for rapid hand task recognition. Nat. Electron. **6**, 64 (2023). <https://doi.org/10.1038/s41928-022-00888-7>
